# Mitochondria targeted nanoparticles for the treatment of mitochondrial dysfunction-associated brain disorders

**DOI:** 10.3389/fbioe.2025.1563701

**Published:** 2025-03-12

**Authors:** Amy Claire Buck, Gerald J. Maarman, Admire Dube, Soraya Bardien

**Affiliations:** ^1^ Division of Molecular Biology and Human Genetics, Department of Biomedical Sciences, Faculty of Medicine and Health Sciences, Stellenbosch University, Cape Town, South Africa; ^2^ South African Medical Research Council Centre for Tuberculosis Research, Division of Molecular Biology and Human Genetics, Faculty of Medicine and Health Sciences, Stellenbosch University, Cape Town, South Africa; ^3^ Centre for Cardio-Metabolic Disease in Africa (CARMA), Division of Medical Physiology, Department of Biomedical Sciences, Faculty of Medicine and Health Sciences, Stellenbosch University, Cape Town, South Africa; ^4^ School of Pharmacy, Faculty of Natural Sciences, University of the Western Cape, Cape Town, South Africa; ^5^ Stellenbosch University Genomics of Brain Disorders Research Unit, Division of Molecular Biology and Human Genetics, Faculty of Medicine and Health Sciences, Stellenbosch University, Cape Town, South Africa

**Keywords:** brain disorders, mitochondrial dysfunction, nanomedicine, mitochondria-targeted nanoparticles, therapy

## Abstract

Mitochondria play a significant role in several cellular activities and their function in health and disease has become an important area of research. Since the brain is a high-energy-demanding organ, it is particularly vulnerable to mitochondrial dysfunction. This has been implicated in several brain disorders including neurodegenerative, psychiatric and neurological disorders, e.g., Parkinson’s disease and schizophrenia. Significant efforts are underway to develop mitochondria-targeting pharmaceutical interventions. However, the complex mitochondrial membrane network restricts the entry of therapeutic compounds into the mitochondrial matrix. Nanoparticles (NPs) present a novel solution to this limitation, while also increasing the stability of the therapeutic moieties and improving their bioavailability. This article provides a detailed overview of studies that have investigated the treatment of mitochondrial dysfunction in brain disorders using either targeted or non-targeted NPs as drug delivery systems. All the NPs showed improved mitochondrial functioning including a reduction in reactive oxygen species (ROS) production, an improvement in overall mitochondrial respiration and a reversal of toxin-induced mitochondrial damage. However, the mitochondrial-targeted NPs showed an advantage over the non-targeted NPs as they were able to improve or rescue mitochondrial dynamics and biogenesis, and they required a lower concentration of the *in vivo* therapeutic dosage of the drug load to show an effect. Consequently, mitochondria-targeted NPs are a promising therapeutic approach. Future studies should exploit advances in nanotechnology, neuroscience and chemistry to design NPs that can cross the blood-brain barrier and selectively target dysfunctional mitochondria, to improve treatment outcomes.

## 1 Introduction

Mitochondrial function is important for numerous cellular activities, including β-oxidation of fatty acids, modification of phospholipids, metabolism of amino acids, the urea cycle, reactive oxygen species (ROS) generation, calcium (Ca^2+^) homeostasis, intracellular signaling pathways, as well as cellular ageing, survival and death (Bagheri-Mohammadi et al., 2023). Disruption of these cellular activities can impair mitochondrial function and contribute to diseases like Parkinson’s, Alzheimer’s, Huntington’s, schizophrenia, and ischemic stroke (Alqahtani et al., 2023; Sultana et al., 2023). While the term “mitochondrial dysfunction” is often misinterpreted or misunderstood, it can be defined as i) the inability of mitochondria to sustain adenosine triphosphate (ATP) production that is sufficient at supporting cellular demands, or ii) the failure of mitochondria to adapt sufficiently to pathologic conditions (Maarman, 2022). Mitochondrial dysfunction is frequently considered to involve collective changes in the electron transfer system (ETS), ROS production, antioxidant enzyme activity, mitochondrial gene/protein expression, mitochondrial membrane potential, and/or fission and fusion rates (Maarman, 2022).

Based on the importance of functional mitochondria and its vulnerability to dysfunction, significant efforts are underway to develop mitochondria-targeting pharmaceutical interventions. However, directly targeting therapeutics to mitochondria is challenging. One of the main barriers to molecules intracellularly reaching mitochondria is its complex membrane network. While therapeutic moieties are capable of passive diffusion through the outer mitochondrial membrane, phospholipid cardiolipin and the highly negative membrane potential make it nearly impossible for therapeutics to pass through the other mitochondrial membranes (Abdel-Mageed et al., 2021). Other disadvantages limiting the effect of some drugs include their poor stability and bioavailability, low solubility, and non-selective biodistribution.

Nanoparticles (NPs) present a novel solution to overcome several of these challenges by improving the bioavailability and enhancing the efficacy of therapeutic moieties (Abdel-Mageed et al., 2021). Notably, while many diverse types of NPs exist, all can be designed to target organelles including mitochondria, thereby facilitating precise disease treatment. In this mini-review, we discuss features of mitochondrial dysfunction in brain disorders and provide a detailed overview of studies that have investigated the treatment of mitochondrial dysfunction using targeted and non-targeted NPs as a drug delivery system. The goal of this mini-review is to highlight the potential of mitochondria-targeted NPs for the treatment of brain disorders, while emphasizing that several knowledge gaps need to be explored before the field is ready for translational medicine applications.

## 2 Features of mitochondrial dysfunction in brain disorders

The regulation and maintenance of cellular metabolism are a critical challenge for the nervous system. Due to the highly complex morphology of neurons, their regulated transmembrane ion gradients and the constant activity of synapses, cellular metabolism of performing and maintaining neural functions is excessively high (Rangaraju et al., 2019). With the human brain being one of the most energy-demanding organs, accounting for 20% of the body’s total energy expenditure (Rangaraju et al., 2019), it is to be expected that mitochondrial dysfunction is implicated in the pathogenesis of several brain disorders (Clemente-Suárez et al., 2023).

The exact roles that mitochondria play in these disorders varies, however, evidence suggests that the progression of brain diseases and mitochondria dysfunction have an interchanging relationship. For example, mitochondrial DNA mutations that disrupt normal cellular function have been implicated in neurodegenerative disease development (Clemente-Suárez et al., 2023). Other examples include neuronal damage and cell death caused by abnormal mitochondrial dynamics, reduced neuronal viability caused by Ca^2+^ dysregulation, and neuronal damage and neurodegeneration because of excessive ROS production and redox homeostasis imbalance (Clemente-Suárez et al., 2023).

Other cases where brain disorders can exhibit mitochondrial dysfunction include inflammatory mediator-induced mitochondrial dysfunction resulting from the production of nitric oxide (NO) and ROS causing damage to mitochondrial lipids, proteins, and DNA (Clemente-Suárez et al., 2023; Missiroli et al., 2020). Moreover, inflammatory signaling pathways can activate pro-apoptotic proteins, leading to mitochondrial outer membrane permeabilization and subsequent release of cytochrome *c* which further propagates the inflammatory response (Clemente-Suárez et al., 2023; Teleanu et al., 2022). Alterations in Ca^2+^ homeostasis due to inflammation can also promote mitochondrial fragmentation via mitochondrial dynamic disruption (Clemente-Suárez et al., 2023). For example, Ca^2+^ influx into mitochondria can trigger excessive mitochondrial fission, impairing mitochondrial function and energy deficits. Other evidence suggests that mitochondrial dysfunction can influence neurotransmitter metabolism and signaling in psychiatric disorders (Daniels et al., 2020). More specifically, the balance of neurotransmitters can be disrupted through multiple mechanisms, such as impaired oxidative phosphorylation (OXPHOS) and reduced ATP production which affects the synthesis, release and reuptake of neurotransmitters such as serotonin, dopamine, and glutamate (Teleanu et al., 2022).

Given the critical role of mitochondria in brain disorders, many therapeutic strategies have been explored to identify biomarkers and protect and restore mitochondrial function (Clemente-Suárez et al., 2023). Mitochondrial protective agents, such as antioxidants and mitochondrial-targeted compounds, have shown promise in preclinical and clinical studies (Jiang et al., 2020). Moreover, recent advances in nanotechnology have opened possibilities for restoring mitochondrial function in brain diseases. Making use of nanomaterials and nanotechnology as novel therapeutic approaches to protect mitochondrial health can improve treatment strategies in brain disorders. With this approach, knowledge of underlying mechanisms of mitochondrial dysfunction can be explored and possible therapeutics can be developed to repair mitochondrial defects and mitigate disease progression while improving patient outcomes.

## 3 How nanomedicine can be used to treat brain disorders

Nanoparticles (NPs) have a comprehensive range of applications and are multifunctional depending on their size and surface functionalities (Najahi-Missaoui et al., 2020). NPs typically range between 1 and 100 nm in size, in at least one dimension and are classified into three classes (Najahi-Missaoui et al., 2020) (Supplementary Figure S1). They can be used to achieve a wide range of medical benefits such as prevention and treatment by utilizing them as a drug delivery system (Abdel-Mageed et al., 2021) (Supplementary Material). For therapeutic applications, NPs require specific characterizations, for example,; size determines the interaction between the NP and biological systems (Hoshyar et al., 2016), and surface charge indicates NP stability and absorption into the cell membrane (Abdel-Mageed et al., 2021; Joudeh and Linke, 2022) (Supplementary Material). Drug loading of NPs can be achieved via adsorption/absorption through drug incubation or by chemical conjugation (Singh and Lillard, 2009; Zashikhina et al., 2023) (Supplementary Material). By applying functionalized groups on the surface of NPs, their application in drug delivery can be personalized according to disease-specific needs by targeting specific cells and intracellular compartments, giving them an advantage over current therapeutic moieties (Kumar et al., 2023). Additionally, nanomedicine has the capability of overcoming the disadvantages of therapeutic moieties by improving drug stability, absorption across biological membranes and consequently bioavailability, targeting intracellular components such as the nucleus, lysosomes or mitochondria, enhancing efficacy, and extending half-life, and reducing cytotoxicity (Abuelezz et al., 2020). Moreover, nanomedicines are actively able to co-target therapeutic moieties to the brain parenchyma and brain lesions by crossing the blood-brain barrier (BBB) and entering cells and intracellular compartments to achieve the delivery of multiple cargos with therapeutic, diagnostic, and theranostic properties (Faria et al., 2023).

Consequently, nanomedicines are becoming more accessible in the market and others in various stages of clinical translation (Shan et al., 2022). To date, there are ∼100 nanomedicines on the market and 563 in clinical process or other stages for a variety of diseases (Shan et al., 2022). However, most of these nanomedicines are focused on cancers and infectious disease treatments with very few for brain disorders (Shan et al., 2022).

Globally, neurological conditions are the leading cause of ill health and disability according to data from the World Health Organization (WHO) and the 2021 Global Burden of Disease, Injuries, and Risk Factor Study (GBD) (Faria et al., 2023; Markowicz-Piasecka et al., 2022; Steinmetz et al., 2024). At present, there are several treatment strategies that target mitochondrial dysfunction in brain disorders including pharmacological treatments, mitotherapy (transplantation/transfer of mitochondria), photobiomodulation and gene therapy (Alshial et al., 2023). While there has been significant effort in the field of mitochondrial medicine, the development of mitochondria-targeted NP therapeutics for brain disorders is hindered. This is due to several translational challenges such as the genetic complexity of brain disorders and of the mitochondrion itself, as well as the heterogeneous clinical presentations of patients (Alshial et al., 2023). Currently, there are four Food and Drug Administration (FDA)-approved nanocrystals for the treatment of schizophrenia (Jia et al., 2023), however, no mitochondria-targeting specific NPs are on the market or in clinical translation. Factors which hinder the clinical application of NPs include the enhanced permeability and retention discrepancies between animal models and humans, unclear *in vivo* fate, and NP toxicity (Jia et al., 2023). The substantial properties of NPs in not only improving the delivery of biopharmaceuticals (Jia et al., 2023), but also its ability to personalize disease treatment with surface modifiers has made them an attractive therapeutic option for the treatment of brain disorders.

## 4 Summary of literature using nanomedicine approaches to treat mitochondrial dysfunction-associated brain disorders

To identify and summarize published work in the area concerning NPs and mitochondrial dysfunction in brain disorders to date, we conducted a literature search of the PubMed database (on 12 December 2023). The following search terms were used: “nanoparticles” AND “mitochondrial” AND “dysfunction” AND “brain” AND “disorders”, and 80 articles were retrieved. After all review articles, book chapters, and other nonrelevant literature were removed and a date selection of the past decade (2013–2023) was applied, 52 articles were returned. These articles were further filtered by removing all articles that did not focus on the terms: “mitochondria dysfunction” or “brain disorders”. After all filters were applied, 36 original research articles were identified and summarized into two tables (Tables 1, 2). More *detailed versions of these tables*, which include NP characterization and properties of the loaded drug, are provided in Supplementary Tables S1, S2, respectively.

**TABLE 1:** *In vitro* and *in vivo* studies of non-targeted nanoparticles and their effect on mitochondrial functioning.

	Type of NP	Drug loaded	Brain disorder	Experimental model and treatment conditions		Effect of the NP treatment on mitochondrial functioning		References^a^
1	Solid lipid NPs (SLN) (C-SLN)	Curcumin	Huntington’s disease (HD)	3-NP-induced HD female Wistar rats		Improved • SDH, NADH dehydrogenase, cyt *c* oxidase, SOD, and mitochondrial F_1_F_0_ ATP synthase activity • Cyt *a*, *b*, and *c* and GSH levels	Reduced • 3-NP-induced mitochondrial swelling • MDA and carbonyl levels • ROS production	Sandhir et al. (2014)
2	Poly (butyl cyanoacrylate) NPs coated with polysorbate 80 (P80-VIP-NPs)	Vasoactive Intestinal Peptide	Parkinson’s disease (PD)	6-OHDA-induced SH-SY5Y cell line		Rescued • Decrease in MMP associated with cell apoptosis		Xu et al. (2015)
3	Glyceryl monoleate NPs, coated with surfactants PF-68 and vitamin E-TPGS (CPNP)	Curcumin	Parkinson’s disease (PD)	Rotenone-treated PC12 and SH-SY5Y cells		Improved • Bcl-2/BAX ratio	Reduced • Cleaved caspase-3 and cleaved PARP expression in rotenone exposed cells	Kundu et al. (2016)
4	SLNs	Thymoquinone (TQ)	Huntington’s disease (HD)	3-NP-treated male albino rats		Improved • SDH enzyme activity	Reduced • Oxidative stress markers (LPO, protein carbonyls and NO)	Ramachandran and Thangarajan (2016)
5	Albumin-based NPs (R-FP-NP)	R-Flurbiprofen	Alzheimer’s disease (AD)	*In vivo* Adult male C57BL/6 mice	*In vitro* CHO cells stably transfected with mouse Ab precursor protein 695 (CHO-APP695)	Improved • Basal respiration • Basal and ATP-linked OCR • Proton leak • Coupling efficiency values • Reserve respiratory capacity • Maximal mitochondrial respiration upon FCCP stimulation	Reduced • Non-mitochondrial respiration following rotenone + antimycin A addition	Wong and Ho (2017)
6	AuNPs	Not stated	Alzheimer’s disease (AD)	Intracerebroventricular-STZ injected Wistar male rats		Induced • Mitochondrial functionality • Proton gradient by succinate	Reduced • Superoxide and NO_2_^−^ levels, and H_2_O_2_ levels indicating ROS reduction	Muller et al. (2017)
7	SLNs	Resveratrol	Vascular dementia (VaD)	Hypoxia-induced oxidative stress BCCAO male Sprague-Dawley (SD) rats		Improved • Levels of GSH • Redox ratio • Nrf2 mRNA expression and protein levels (nuclear to cytoplasmic ratio) Rescued • Mn-SOD activity	Reduced • Production of ROS and LPO •·Mitochondrial GSSG and protein carbonyl levels	Yadav et al. (2018)
8	PEG-PLGA NPs	Curcumin	Cerebral ischemia-reperfusion (CIR)	CIR-induced female SD rats		Improved • Mitochondrial membrane microviscosity • Cytosolic Bax pool values • Cyt *c* levels for the mitochondrial fractions Rescued • Bcl-2 expression to normal cellular level • iNOS and COX-2 expression levels	Reduced • ROS levels • NADH oxidase activity • Cyt *c* in cell cytosol • Level of the apoptotic factor in CIR-induced rats Prevented • CIR induced lipid peroxidation • Mitochondrial Bax accumulation • Activation of caspase-3 and -9	Mukherjee et al. (2019)
9	Fe_3_O_4_ magnetic NPs	Curcumin	Schizophrenia (SCZ)	Cerebellum cells of ketamine-treated Wistar rats		Improved • Complex II activity (measured by SDH activity) • MMP • ATP levels	Reduced • ROS production in brain mitochondria • Cyt *c* release	Naserzadeh et al. (2018)
10	NPs synthesised using biocompatible polymers as the antisolvent (NRSV)	Resveratrol	Parkinson’s disease (PD)	Rotenone-treated male albino Wistar rats		Rescued • Complex I activity • SOD, CS, and aconitase activities	Reduced • Rotenone-induced lipid peroxidation	Palle and Neerati (2018)
11	Cu_x_O NP clusters (NCs)	Not stated	Parkinson’s disease (PD)	MPP^+^-treated SHSY-5Y cells, UVA-treated NIH-3T3 cells & OxLDL-treated cultured PIE cells		Improved • Cell viability in all cellular models	Reduced • Caspase-3 protein expression in all cellular models	Hao et al. (2019)
12	AuNPs associated with NAC	Not stated	Sepsis-induced brain injury	CLP-induced adult male Wister rats		Improved • Complex I activity	Reduced • LPO in the hippocampus and prefrontal cortex • Protein carbonyls in the hippocampus	Petronilho et al. (2020)
13	Palladium hydride (PdH) NPs	Not stated	Alzheimer’s disease (AD)	*In vivo* 3×Tg-AD mice carrying human gene mutants APPswe, PS1M146V and tauP301L	*In vitro* Over expressed human Swedish mutant APP695 cell lines (N2a-SW)	*In vivo* Reduced • Aβ oligomers levels Rescued • Drp1 and Mfn2 expression levels Improved • COX IV expression	*In vitro* Improved • Basal respiration, ATP production, H^+^ proton leak and max respiration • COX IV expression Rescued • Drp1 and Mfn2 expression levels • MMP Reduced • Ca^2+^ level	Zhang et al. (2019)
14	Cerium oxide NPs (CeONPs)	Not stated	Mild traumatic brain injury (mTBI)	*In vitro* Mild lateral fluid percussion brain injury induced adult male SD rats	*In vivo* Mixed organotypic neuronal cultures from 94A Cell Injury Controller neonatal rats	*In vivo* Improved • SOD and activity • GSH/GSSG ratio • NT levels to normal Reduced • LOOH levels	*In vitro* Restored • Response to injury-induced glutamate signalling	Bailey et al. (2020)
15	AuNPs	Not stated	Alzheimer’s disease (AD)	Aβ_1-42_-treated hNSCs		Improved • PGC1α, NRF-1, and Tfam mRNA levels • ATP levels and mitochondrial mass	Normalised • Max mitochondrial respiratory function • Cyt *c* oxidase activity • MMP and mitochondrial morphology	Chiang et al. (2020)
16	AuNPs	Not stated	Alzheimer’s disease (AD)	OA-treated Wistar male rats		Rescued • Mitochondrial activity (especially complex V)	Reduced • Succinate-induced H_2_O_2_ production	dos Santos Tramontin et al. (2020)
17	Zinc Oxide (ZnO) NPs	Not stated	Neuronal differentiation	NGF differentiated PC12 cells		Improved • Basal respiration	Reduced • Maximal respiration • ATP production and spare respiratory capacity • Proton leak	Srivastava et al. (2020)
18	Nanocrystals (HstN)	Hesperetin	Alzheimer’s disease (AD)	SH-SY5Y cell line harbouring neuronal amyloid precursor protein (APP695)		Improved • Complex I and IV activity, OXPHOS, ETC., and leak respiration • ATP levels • *CS*, *COX5A*, *NDUFV1*, and *ATP5F1D* mRNA levels • Aβ_1-40_ expression	Reduced • Peroxidase and cyt *c* activity • Small lowering effect on ROS	Babylon et al. (2021)
19	Borneol modified octahedral palladium nanocomposites (Pd@PEG@Bor)	Not stated	Alzheimer’s disease (AD)	*In vitro* H_2_O_2_/Aβ -treated SH-SY5Y cells for ROS and MMP detection, respectively	*In vivo* C57BL/6 and 3XTg-AD mice	Recovered • Low MMP in Aβ-treated SH-SY5Y cells	Reduced • OH^−^ levels at higher concentrations of Pd@PEG@Bor • Aβ-induced cell apoptosis	Jia et al. (2021)
20	Nanoemulsions	Curcumin	Parkinson’s disease (PD)	Rotenone-treated Male Swiss Albino mice		Prevented • Rotenone-induced inhibition of mitochondrial complex I activity		Ramires Júnior et al. (2021)
21	AuNPs	Not stated	Hypercholesterolemia	Adult Swiss mice fed a high cholesterol diet		Improved • Prefrontal cortex complex I activity in both normal and high cholesterol diet		Rodrigues et al. (2021)
22	SLNs (GSE/DA-SLN)	Grape seed extract and dopamine	Parkinson’s disease (PD)	OECs and 6-OHDA-induced SH-SY5Y cells		Improved • Antioxidant activity		Trapani et al. (2021)
23	Polydopamine NPs (PDNPs)	Not stated	Autosomal recessive spastic ataxia of Charlevoix-Saguenay (ARSACS)	TBH-treated ARSACS patient-derived fibroblasts and healthy fibroblasts		Recovered • TBH-induced elongation loss of the mitochondria Prevented • Increment in apoptotic and necrotic cells • TBH-induced fragmentation of the mitochondria and mitochondrial damage through MMP stability	Reduced • Basal ROS	Battaglini et al. (2022)
24	AuNPs	Glutathione	Alzheimer’s disease (AD)	Aβ_1-42_-treated hNSCs		Improved • SOD, catalase and HO^−1^ activity • PGC1α, NRF-1 and Tfam protein levels • MMP Normalized ·Oxidative stress in Aβ-induced cells • Release of cyt *c* from the mitochondria into the cytosol	Reduced • Superoxide production • Cyt *c* oxidase activity Rescued • Aβ-induced ATP loss, D-loop and mitochondrial mass • Basal respiration, ATP-linked respiration, and maximal respiration capacity • PGC1α, NRF-1 and Tfam gene expression	Chiang and Nicol (2022)
25	PLGA-based NPs	PINK1 siRNA	Photothrombosis-induced ischemic stroke (PTS)	RB-induced PTS murine model		Reduced • Mitophagy and autophagy prior to PTS induction via the reduction of PINK1, LC3B, and lamp1 protein expression • MMP depolarization		Choi et al. (2023)
26	β-cyclodextrin NPs (NSβ-CD)	Not stated	Niemann-Pick type C1 (NPC)	Blood and urine samples from patients with NPC and fibroblasts derived from skin biopsies taken from patients with NPC		Reduced • Mitochondrial superoxide production		Hammerschmidt et al. (2023)
27	Tween 80 coated PLGA NPs	NONOates	Alzheimer’s disease (AD)	LPS-induced adult Swiss Abino mice		Improved • Cyt *c* oxidase enzyme activity • ATP levels		Samir et al. (2023)

^a^ Articles arranged in chronological order.

3-NP, 3-Nitropropionic Acid; 6-OHDA, 6-hydroxydopamine; ATP, adenine triphosphate; ATP5F1D: ATP, synthase F1 subunit delta; BAX, Bcl-2-associated X-protein; Bcl-2, B-cell leukemia/lymphoma 2 protein; CLP, cecal ligation and puncture; COX IV, Cytochrome c oxidase subunit IV; COX-2, Cyclooxygenase-2; COX5A, Cytochrome c oxidase subunit 5A; CS, citrate synthase; Cyt a, b, c, Cytochrome a, b, c; Drp1, Dynamin related protein 1; ETC., electron transfer chain; FCCP, Carbonylcyanide-p-trifluoromethoxyphenylhydrazone; GSH, glutathione; GSSG, oxidised glutathione; H_2_O_2_, hydrogen peroxide; iNOS, nitric oxide synthase; lamp1, Lysosomal associated membrane protein 1; LC3B, Light chain 3 beta; LOOH, lipid hydroperoxide; LPO, lipid peroxidation; LPS, lipopolysaccharide; MDA, malondialdehyde; Mfn2, Mitofusin-2, protein; MMP, mitochondrial membrane potential; Mn-SOD, manganese superoxide dismutase; MPP^+^, 1-methyl-4-phenylpyridinium; mRNA, messenger ribonucleic acid; Mt, Mitochondria; NADH, nicotinamide adenine dinucleotide; NDUFV1, NADH:ubiquinone oxidoreductase core subunit V1; NGF, nerve growth factor; NO, nitric oxide; NO_2_^−^: nitrite; NP(s), Nanoparticle(s); NRF1/2, Nuclear factor erythroid 2-related factor 1/2; NT, nitrotyrosine; OCR, oxygen consumption rate; OxLDL, Oxidised low-density lipoprotein; OXPHOS, oxidative phosphorylation; PARP, Poly (ADP-ribose) polymerase; PF-68, Pluronic F-68; PGC1α, Peroxisome proliferator-activated receptor gamma coactivator 1-alpha; PINK1, PTEN, induced kinase 1; RB, retinoblastoma; R-FP, R-Flurbiprofen; ROS, reactive oxygen species; SDH, succinate dehydrogenase; SOD, superoxide dismutase; STZ, streptozotocin; TBH, Tert-butylhydroperoxide; Tfam, Mitochondrial transcription factor A; TPGS, tocophersolan; UVA, Ultra-violet A; VIP, vascular intestinal peptide.

**TABLE 2:** *In vitro* and *in vivo* studies of mitochondria-targeted nanoparticles and their effect on mitochondrial functioning.

	Type of NP	Drug loaded	Mt targeting surface modifier	Brain disorder	Experimental model and treatment conditions^a^		Effect of the NP treatment on mitochondrial functioning		References^b^
1	Ceria NPs	Ceria	TPP^+^	Alzheimer’s disease (AD)	*In vivo* 5XFAD transgenic AD mice	*In vitro* Aβ-induced SH-SY5Y cells	*In vivo* Recovered Severe cristae disruptions in 5XFAD mice Reduced 4-HNE in 5XFAD mice	*In vitro* Prevented Aβ-induced mitochondrial ROS	Kwon et al. (2016)
2	4 hydroxyl-terminated PAMAM dendrimers	D-NAC	TPP^+^	Traumatic brain injury (TBI)	Human macrophages, murine microglia and paediatric TBI model		Rescued • Mitochondrial O_2_^−^ levels back to within 10% of healthy control cells • MMP to approximately 90% of healthy cell levels	Reduced O_2_^−^ levels compared to H_2_O_2_ stimulated cells Preserved Metabolic activity of H_2_O_2_ stimulated cells	Sharma et al. (2018)
3	Red blood cell (RBC) membrane-camouflaged human serum albumin NPs	Curcumin and GSH	TPP^+^	Alzheimer’s disease	*In vivo* Male ICR mice and Sprague-Dawley (SD) rats treated with OA	*In vitro* RAW264.7, BMECs and HT22 cells	Reduced • Mitochondrial ROS levels *in vivo* and *in vitro* • SOD, ϒ -GT, MDA and H_2_O_2_ levels *in vitro* • Cell apoptosis rate *in vitro*		Gao et al. (2020)
4	RBC membrane-coated nanostructured lipid	Resveratrol (RSV)	TPP^+^	Alzheimer’s disease	*In vivo* Male ICR mice, SD rats, and APP/PS1 mice	*In vitro* Peritoneal macrophages and primary astrocytes isolated from C57BL/6J mice, Aβ-treated bEnd.3 and HTT2 cells	*In vivo* Reduced Mitochondrial ROS in APP/PS1 mice	*In vitro* Reduced Mitochondrial ROS, MnSOD, MDA and cell apoptosis in Aβ-treated HTT2 cells	Han et al. (2020)
5	PLGA-b-PEG-TPP	ARVs/cART	TPP^+^	HIV-associated neurocognitive disorders	*In vivo* EcoHIV and Meth infected Balb/c albino female mice and C57BL/6 male mice	*In vitro* HMC3, CRL-3304, and nHA cells and isolated astrocytes and neuronal cells	Rescued Mitochondrial ROS in HIV + Meth induced microglia cells Improved ATP production	Reduced • O_2_^−^ levels • Oxidative stress (measured by the mRNA levels of the ROS markers GCLC, GCLM, and GPX7) in HIV + Meth induced cells ·ROS levels in the mice	Surnar et al. (2021)
6	AuNPs	Au	Hypericin	Glioblastoma (GBM)	HEK293T, LN18 and C6 cell lines		Induced • MMP depolarisation and loss • Mitochondria intracellular ROS apoptosis • ROS-induced glioma cell death and overexpression of caspase-3, therefore, abrogating GBM proliferation		Kaundal et al. (2022)
7	Citraconylation modified PEG	Hybrid peptide HNSS	SS31 targeting moiety and humanin	Alzheimer’s disease	3xTg-AD transgenic mice with mutated human APP(Swe), tau (P301L), and PS1(M146 V) genes		Prevented Intracellular and mitochondrial ROS production Rescued ·GSH and SOD activity • Aβ_25−35_-induced impaired mitochondrial function • Morphology of mitochondria • Dynamic imbalance of MFN1 and DRP1 proteins	Improved • p-STAT3, cyt *c* MTCO1 (subunit of complex IV protein) and MnSOD expression levels • p-STAT3/STAT3 ratio • TAOC and SOD activity • PGC-1α mRNA level Reduced MDA levels	Qian et al. (2022)
8	Tetrahedral DNA framework-based NPs	Functional antisense oligonucleotide (ASO)	TPP^+^	Alzheimer’s disease	*In vivo* 3xTg-AD model mice	*In vitro* Aβ-treated SH-SY5Y cells and HEK293/TAU cells	*In vivo* Rescued Neuronal mitochondria by ASO-induced miRNA-24a knock down	*In vitro* Rescued Apoptosis of Aβ-treated SH-SY5Y cells Improved MMP in Aβ-treated SH-SY5Y cells	Li et al. (2023)
9	Cu_2_-xSe-based NPs	Curcumin	TPP^+^	Parkinson’s disease	*In vivo* MPTP and US-treated C57BL/6 mice	*In vitro* RAW 264.7 and MPTP induced SH-SY5Y cell lines	*In vivo* Improved PGC-1*α*, SIRT1 TFAM, and Gpx4 expression Rescued Ndufs1 expression and Gpx4 enzyme activity in mitochondria	*In vitro* Improved • PGC-1*α* deacetylation and PGC-1*α*-induced PPAR*γ*, NRF1, and TFAM mRNA levels • NAD^+^/NADH ratio, SIRT1 activation and mtDNA copy number Rescued Gpx4, CAT, and SOD2 levels, GSH/GSSG ratio and Mitochondrial cristae Prevented MPP^+^-induced mitochondrial ROS production and reduction of MMP Reduced Cyt *c* release	Zheng et al. (2023)

^a^ Experimental models relating only to mitochondrial studies are mentioned.

^b^ Articles arranged in chronological order.

4-HNE, 4-Hydroxynonenal; 5XFAD, Mice that bear five Familial Alzheimer’s Disease-linked mutated genes; ARVs/cART, Antiretrovials/combined antiretrovirals; ASO, antisense oligonucleotide; ATP, adenosine triphosphate; Au: Gold; Aβ, amyloid beta peptide; CAT, catalase; Cu_2_-xSe, Copper chalcogenide; cyt, Cytochrome; DNA, deoxyribonucleic acid; D-NAC, Dendrimer conjugated N-acetyl cysteine; DRP1, Dynamin related protein 1; GCLC, Glutamate-cysteine ligase catalytic subunit; GCMC, Glutamate-cysteine ligase modifier subunit; Gpx4, Glutathione peroxidase 4; GPX7, Glutathione peroxidase 7; GSH, glutathione; GSSG, oxidised glutathione; H_2_O_2_, hydrogen peroxide; HIV, human immunodeficiency virus; MDA, malonaldehyde; Meth, Methamphetamine; MFN1, Mitofusin 1; MMP, mitochondrial membrane potential; MnSOD, manganese superoxide dismutase; MPTP, 1-methyl-4-phenyl-1, 2,3,6-tetrahydropyridine; mRNA, messenger ribonucleic acid; MTCO1, Mitochondrially Encoded Cytochrome C Oxidase I; mtDNA, Mitochondrial DNA; NAD^+^, nicotinamide adenine dinucleotide; NADH, Nicotinamide adenine dinucleotide + hydrogen; Ndufs1, NADH:ubiquinone oxidoreductase core subunit S1; NP(s), Nanoparticle(s); NRF1, Nuclear respiratory factor 1; O_2_^−^, superoxide anion; OA, okadaic acid; PAMAM, polyamidoamine; PGC1α, Peroxisome proliferator-activated receptor gamma coactivator 1-alpha; PLGA-b-PEG, Poly (D,L-lactic-co-glycolic acid)-block-polyethylene glycol); PPAR*γ*, Peroxisome proliferator-activated receptor gamma; (p)STAT3, (Phosphorylated) transducer and activator of transcription-3; ROS, reactive oxygen species; SIRT1, Sirtuin 1; SOD, superoxide dismutase; SOD2, Superoxide dismutase 2; SS31, Szeto-Schiller peptide 31; TAOC, total antioxidant capacity; Tfam, Mitochondrial transcription factor A; TPP^+^, triphenylphosphonium cation; US, ultrasonic; ϒ-GT, Gamma-glutamyl transpeptidase.

### 4.1 Studies that used non-targeted nanoparticles

Of the 36 research articles, 27 used NPs that did not target mitochondria and are summarized in Table 1 with a more detailed version in Supplementary Table S1. In the non-targeted NP studies, a wide variety of NP types were used. The choice of the type of NP intended for use in drug delivery systems is based on the physiochemical properties, characteristics, benefits and applications of the NP type. These include size, shape, surface functionalization, core stability, biocompatibility, release kinetics, targeting, and accumulation (Yusuf et al., 2023).

Across the studies, a common improvement was reported in; i) mitochondrial respiration particularly in basal respiration, complex I activity, ATP levels, and leak respiration, and ii) the reduction in ROS production (5, 18, 20, 23, 26 in Table 1; Supplementary Table S1). An effect on i) the reduction in superoxide and hydrogen peroxide levels indicating a lowering of ROS production, ii) the improvement in ATP production, PGC1α, NRF-1 and Tfam mRNA levels, thereby indicating an improvement in OXPHOS and mitochondrial biogenesis, and iii) the normalization of the mitochondrial membrane potential and cytochrome *c* oxidase activity was also reported (6, 12, 13, 15, 16, 19, 21, and 24 in Table 1; Supplementary Table S1). Lastly, i) an improvement in complex II activity, mitochondrial membrane potential and ATP levels, ii) a reduction in brain mitochondrial ROS production and cytochrome *c* release, iii) an improvement in basal respiration, superoxide dismutase (SOD) activity, and catalase activity, indicating a decrease in ROS production (Saxena et al., 2023), iv) a reduction in proton leak and caspase-3 protein expression, indicating a lowering of apoptotic activation and prevention of cell atrophy (Brentnall et al., 2013), and v) the restoration of the response to injury-induced glutamate signaling, implying an improvement of OXPHOS and glycolysis (Hillen and Heine, 2020) (9, 11, 14, 17–20, 22, 23 in Table 1; Supplementary Table S1), was observed.

Although the function of the NPs in these studies was not to specifically target mitochondria, they all proved to affect mitochondria functioning. The two most common drugs used in these studies were curcumin (1, 3, 8, 9, and 20 in Table 1) and resveratrol (7 and 10 in Table 1). Curcumin and resveratrol are strong antioxidants, and this property is evident with the NPs loaded with these therapeutic moieties showing a marked improvement in antioxidant functioning and reduction in ROS production.

The concentration of the therapeutic moieties loaded in/on the NPs, however, should be noted. The therapeutic dosage of the two common drugs in Table 1, specifically *in vivo* studies, ranges from 5–50 mg/kg body weight and 10–40 mg/kg body weight for curcumin and resveratrol, respectively. These values far exceed the Allowable Daily Intake (ADI) of both drugs, which could lead to cellular toxicity. According to the European Food Safety Authority (EFSA) reports, the ADI of curcumin is 0–3 mg/kg body weight (Hewlings and Kalman, 2017) and the ADI of resveratrol is 7.5 mg/kg body weight (Louis Bresson et al., 2016).

### 4.2 Studies that used mitochondria-targeted nanoparticles

The mitochondrial membrane complex structure and its highly negative membrane potential inhibit the entry of anionic drugs and molecules into the mitochondrial matrix (Tabish and Hamblin, 2021). Non-targeted NPs enter the cell via the endocytosis process through the direct coating of the NPs with plasma proteins, which allows entry of the NPs through the plasma membrane (Oh and Park, 2014). However, due to the plasma membrane being selectively permeable, entry of the NPs into the cells is size, shape and surface chemistry dependent, which ultimately determines their systemic elimination and toxicity (Oh and Park, 2014). Consequently, the need to design and develop target-specific NPs is of growing interest. Of the 36 research articles, nine used mitochondria-targeted NPs and are summarized in Table 2 and Supplementary Table S2.

There are currently two approaches to achieve mitochondrial targeting of NPs: active and passive targeting. Active targeting involves the surface functionalization of NPs with lipophilic cations (mitochondrial-specific ligands), while passive targeting involves the tuning of the NP characteristics to improve their specificity (Tabish and Hamblin, 2021). There are a variety of mitochondrial-specific moieties that can be attached to either drug-loaded or unloaded NPs. These ligands include triphenylphosphonium (TPP^+^), tetramethylrhodamine-5-isothiocyanate, mitochondrial peptides, dequalinium, and natural products (such as hypericin and glycyrrhetinic), to name a few (Tabish and Hamblin, 2021). TPP^+^ is a widely studied mitochondrial-targeting ligand used in the preparation of non-toxic NPs. The conjugation of TPP^+^ to NPs increases the lipophilicity and enables the NPs to freely cross the mitochondrial membrane bilayers (Tabish and Hamblin, 2021).

The common effects of *in vivo* studies include; i) the normalization of superoxide levels, SOD and GSH activity, imbalance of MFN1 and DRP1, and the mitochondrial membrane potential and mitochondrial morphology, indicating the normalization of OXPHOS and ROS levels as well as mitochondrial dynamic balance, ii) reduction in ROS production and MDA levels, and iii) the improvement of PGC-1α levels, SOD activity, and ATP production, suggesting an increase in mitochondrial biogenesis, ROS reduction and OXPHOS (2, 5 and 7 in Table 2; Supplementary Table S2). Other common effects *in vivo* and *in vitro* are the reduction in ROS, MDA and SOD activity, and cell apoptosis (3 and 4 in Table 2; Supplementary Table S2). The common effects of the study which aimed to induce glioma cell death include the induction of mitochondrial membrane depolarization, ROS production, caspase-3 overexpression and apoptosis *in vitro*. These results indicate the successful abrogation of glioblastoma proliferation through the activation of mitochondrial destruction, thereby highlighting the dual property of organelle-targeted NPs (6 in Table 2; Supplementary Table S2). The other effects included apoptotic rescue and the improvement in mitochondrial membrane potential (8 in Table 2; Supplementary Table S2).

As with the studies in Table 1, the most common drug used in these studies was curcumin (3 and 9 in Table 2). However, in contrast to the *in vivo* therapeutic dosage of curcumin seen in Table 1. (5–50 mg/kg body weight), the concentrations of curcumin *in vivo* in Table 2, were much lower (2 and 5 mg/kg body weight). While this concentration range is still higher than the ADI of curcumin (0–3 mg/kg body weight (Hewlings and Kalman, 2017), the dosage is significantly less than those used in non-targeted NPs. This is important as it highlights that targeted NPs decrease the therapeutic threshold dosage, meaning that lower therapeutic concentrations are needed to provide optimal treatment outcomes while minimizing side effects (Tian et al., 2022).

Overall, the results of the mitochondrial-targeted NPs demonstrated a direct effect of the NPs on mitochondrial functioning as shown by the recovery/improvement of mitochondrial morphology, and structure as well as crucial proteins involved in mitochondrial maintenance. It is important to note that, unlike the untargeted NPs, mitochondrial-targeted NPs were able to improve or rescue mitochondrial dynamics and biogenesis. The predominant effects of mitochondria-targeted NPs were the recovery of mitochondria cristae, mitochondrial membrane potential, and the overall morphology of mitochondria, the reduction of mitochondrial ROS production, MDA and superoxide levels, and cell apoptosis, as well as the improvement in PGC-1α, SIRT1, and TFAM levels, all of which are necessary for proper mitochondrial functioning.

## 5 Knowledge gaps and future directions

Mitochondria-targeted NPs hold potential for the treatment of various brain disorders, with an illustrated summary of the potential effects mitochondria-targeted NPs have on mitochondrial functioning shown in Supplementary Figure S2. However, several knowledge gaps in this area of research need to be addressed before advancements in the field can be made. While there is evidence to support the implication of mitochondrial dysfunction in brain disorders (as shown in Tables 1, 2), the specific mechanisms that lead to mitochondrial dysfunction need to be further elucidated. Consequently, more research is needed to understand exactly how the ETS of mitochondria and other aspects of mitochondrial function are involved in pathogenesis. This could be done using high-resolution respirometry (HRR), specifically the Oroboros Oxygraph (O2k), to measure mitochondrial respiratory control relative to specific tissue/cell types across various metabolic states (Walsh et al., 2023). Additionally, the design of NPs to specifically cross the BBB and accumulate in mitochondria of brain cells need to be further optimized and developed to produce an efficient drug delivery system. Achieving specificity and selectivity for mitochondria is necessary to avoid off-target effects and potential toxicity. Therefore, a crucial aspect of target-specific NPs is to design NPs that selectively target dysfunctional mitochondria in diseased cells while avoiding healthy mitochondria. Furthermore, while there is promising evidence of NPs from preclinical studies, translating the NP’s effectiveness to clinical settings requires more evaluation of their safety and efficacy *in vivo* and ultimately in human trials. Moreover, additional studies on the long-term effects and stability of NPs need to be performed to assess the feasibility of mitochondria-targeted NPs as therapeutic agents. Factors such as NP degradation, potential accumulation in non-target tissues, and immune response need to be investigated thoroughly.

To address these knowledge gaps and drive the field forward, future research should focus on collaborative interdisciplinary research involving researchers from different fields to develop novel therapeutic mitochondria-targeted NPs. Conducting well-designed clinical trials to evaluate the safety, tolerability, and efficacy of mitochondria-targeted NPs in patients with various brain disorders, while focusing on patient stratification and personalised medicine approaches, are an essential part of the process. By addressing these and other challenges, while exploiting advances in nanotechnology, neuroscience, and medicine, the field of mitochondria-targeted NPs holds great potential for transforming brain disorder treatment and improving patient outcomes in the future.

## 6 Concluding remarks

All the NPs that were tested in the reviewed studies showed improved mitochondrial functioning. These features included a reduction in ROS production, an improvement in overall mitochondrial respiration and a reversal of toxin-induced mitochondrial damage. However, one of the major limitations to these studies was the use of the ROS assay to measure mitochondrial function and dysfunction *in vivo*. While ROS assays are useful for understanding how ROS levels impact health and disease, the use of HRR, such as the Oroboros O2k, can help identify the exact cause of the mitochondrial dysfunction by measuring different components of mitochondrial bioenergetics. This does not only aid in the identification of the underlying mechanisms that cause mitochondrial dysfunction, but may assist with developing more effective treatment modalities. Although the field of nanomedicine shows promise, several technical challenges, as stated above, need to be addressed before this technology can be used to treat patients.

In conclusion, the improvement and refinement of nanotechnologies may play a critical role in the way mitochondrial dysfunction in brain disorders is successfully treated in future, by delivering drugs and other agents directly to the site of the disease. This should lead to less toxicity, better adherence and ultimately improved treatment outcomes.
