# Supplementary material for: Mitochondria targeted nanoparticles for the treatment of mitochondrial dysfunction-associated brain disorders
Source: Front Bioeng Biotechnol. 2025 Mar 12;13:1563701. doi: 10.3389/fbioe.2025.1563701 (PMC11937128; doi:10.3389/fbioe.2025.1563701)
Supplement: Supplementary file 1 [file DataSheet1.docx]

Supplementary Material

Mitochondria targeted nanoparticles for the treatment of mitochondrial dysfunction-associated brain disorders

Amy Claire Buck^1,2^, Gerald Maarman^3^, Admire Dube^4^, Soraya Bardien^1,2,5,*^

^1^Division of Molecular Biology and Human Genetics, Department of Biomedical Sciences, Faculty of Medicine and Health Sciences, Stellenbosch University, Cape Town, South Africa

^2^South African Medical Research Council Centre for Tuberculosis Research; Division of Molecular Biology and Human Genetics, Faculty of Medicine and Health Sciences, Stellenbosch University, Cape Town

^3^Centre for Cardio-Metabolic Disease in Africa (CARMA), Division of Medical Physiology, Department of Biomedical Sciences, Faculty of Medicine and Health Sciences, Stellenbosch University, Cape Town, South Africa

^4^School of Pharmacy, Faculty of Natural Sciences, University of the Western Cape, Cape Town, South Africa

^5^Stellenbosch University Genomics of Brain Disorders Research Unit; Division of Molecular Biology and Human Genetics, Faculty of Medicine and Health Sciences, Stellenbosch University, Cape Town, South Africa

*** Correspondence:**Soraya Bardien
[sbardien@sun.ac.za](mailto:sbardien@sun.ac.za)

Contents

[1. Definition, classification, size, drug loading and characterization of nanoparticles 2](#_Toc192063701)

[2. Supplementary Figures and Tables 3](#_Toc192063702)

[Supplementary Figure S1 3](#_Toc192063703)

[Supplementary Figure S2 4](#_Toc192063704)

[Supplementary Table S1. 5](#_Toc192063705)

[Supplementary Table S2 14](#_Toc192063706)

[3. References 19](#_Toc192063707)

Supplementary Data


# Definition, classification, size, drug loading and characterization of nanoparticles

Nanomedicine is the term used to describe the application of nanocarriers/nanoparticles (NPs) for diagnosis, monitoring, screening, prevention and treatment of diseases to achieve medical benefits (Abdel-Mageed et al., 2021). NPs are typically defined as particles ranging between 1 and 100 nm in size, in at least one dimension (Najahi-Missaoui et al., 2020), although particles with sizes up to 1000 nm are also considered (Zielinska et al., 2020). Typically, NPs can be classified into three classes, namely i) organic, ii) inorganic, and iii) carbon-based (Supplementary Figure. 1) (Joudeh & Linke, 2022).

Drug loading of NPs is achieved using various methods (Singh & Lillard, 2009). Drugs can be incorporated during NP synthesis, via adsorption/absorption through drug incubation with the NP, post-synthesis (Singh & Lillard, 2009), or via chemical conjugation of the drug onto the surface of the NP (Zashikhina et al., 2023). For therapeutic applications, NPs typically require specific characterizations including size, polydispersity index (PDI), surface charge and shape (Abdel-Mageed et al., 2021). The size of NPs determines how they interact with biological systems such as cellular membranes and blood vessels. More importantly, the smaller the NP, the deeper the tissue penetration, clearance avoidance by the liver and spleen, as well as its ability to cross biological barriers such as the blood-brain barrier (BBB) (Hoshyar et al., 2016). Surface charge is measured by zeta potential (ZP) and is an indicator of NP stability and primary absorption of NPs into the cell membrane (Abdel-Mageed et al., 2021; Joudeh & Linke, 2022). Additionally, the surface charge of NPs influences the interaction of the NP with cells. A strong cationic NP interacts more favorably with cell membranes therefore enhancing their cellular uptake however, studies have shown that due to the enhanced uptake of the cationic NP in the cell, they are prone to be more toxic to the cell (Aryal et al., 2019).

# Supplementary Figures and Tables
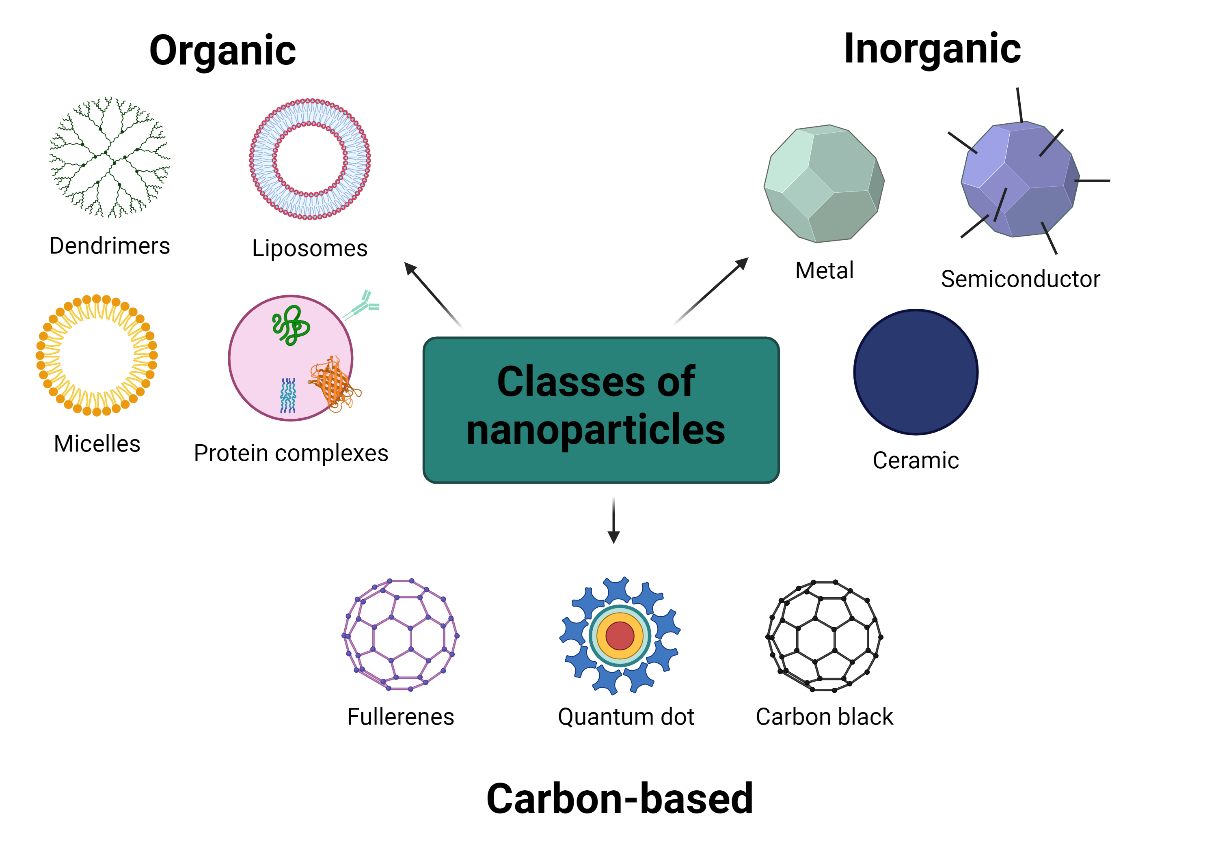


Supplementary Figure S1**.** Nanoparticles can be classified into three classes: Organic, Inorganic, and Carbon-based.


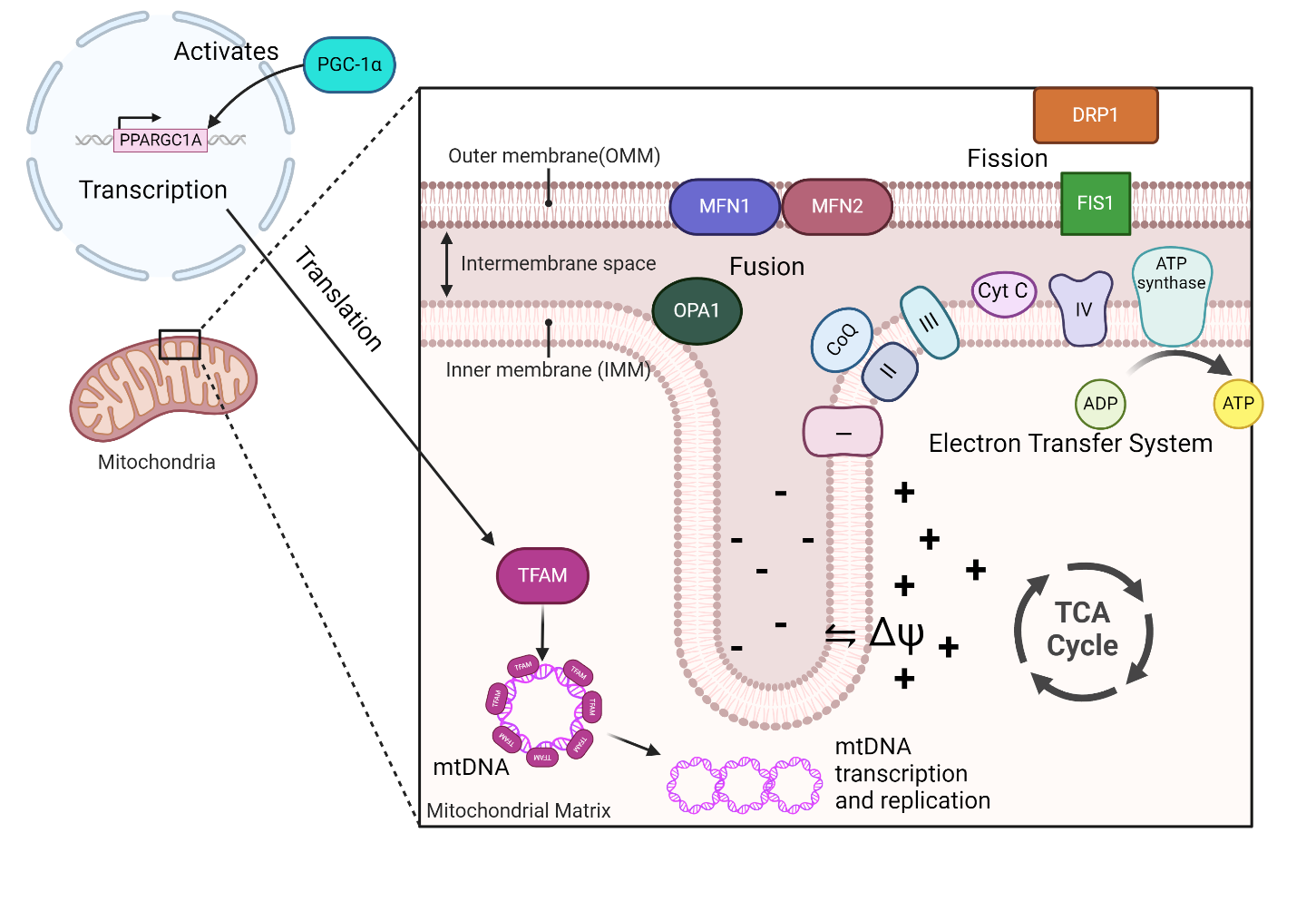


Supplementary Figure S2**.** *A summary of the effects of mitochondria-targeted nanoparticles on mitochondrial function.* The predominant effects of the mitochondria-targeted NPs included: i) the recovery of crucial proteins involved in mitochondrial maintenance such as MFN1/2 and OPA1 (mitochondrial fusion) and DRP1 and FIS1 (mitochondrial fission), ii) the recovery or improvement of the electron transfer system and ultimately oxidative phosphorylation, iii) the recovery of mitochondrial membrane and the overall morphology of mitochondria, and iv) the improvement in PGC-1α and TFAM protein levels which are necessary for proper mitochondrial functioning.

Supplementary Table S1. *In vitro* and *in vivo* studies of drug loaded nanoparticles (NPs) and the effect on mitochondria (mt) functioning in the literature

|  | Type of NP | Characteristics of NP | Drug loaded^[[1]](#footnote-1)^ | Brain disorder | Experimental model  and treatment conditions | | Effect of the NP treatment on Mt functioning | | Reference^[[2]](#footnote-2)^ |
| --- | --- | --- | --- | --- | --- | --- | --- | --- | --- |
| 1 | Solid lipid NPs (SLN) (C-SLN) | **Hydrodynamic diameter (nm)**  **·**148 | **Curcumin:**  **·**Potent natural antioxidant | Huntington’s disease | 3-NP-induced HD female Wistar rats  **·**40 mg/kg b.w by gavage daily for 7 days 1h after 3-NP injection | | **Improved:**  **·**SDH, NADH dehydrogenase, cyt *c* oxidase, SOD, and mitochondrial F1F0 synthase activity  **·**Cyt *a*, *b*, and *c* and GSH levels | **Reduced:**  **·**3-NP-induced mitochondrial swelling  **·**MDA and carbonyl levels  **·**ROS production | (Sandhir et al., 2014) |
| 2 | Poly (butyl cyanoacrylate) NPs coated with polysorbate 80 (P80-VIP-NPs) | **Hydrodynamic diameter (nm)**  **·**Blank-NPs = 119.7 ± 7.3  **·**P80-VIP-NPs = 124.7 ± 8.6  **ζ-potential (ZP) (mV)**  **·**Blank-NPs = −22.0 ± 6.8  **·**P80-VIP-NPs = −12 ± 5.8 | **Vasoactive Intestinal Peptide:**  **·**Stimulates astrocytic mitosis and neurite outgrowth  **·**Prevents excitotoxic glutamate toxicity | Parkinson’s disease | 6-OHDA-induced SH-SY5Y cell line  **·**0,1,10,100 and 1000 nM for 4 h, followed by 50 µM 6-OHDA for 24 h | | **Rescued:**  **·**Decrease in MMP associated with cell apoptosis | | (Xu et al., 2015) |
| 3 | Glyceryl monoleate NPs, coated with surfactants PF-68 and vitamin E-TPGS (CPNP) | **Hydrodynamic diameter** **(nm)**  **·**93 ± 11  **ZP (mV)**  **·**CPCP = −30.9 ± 0.88 | **Curcumin:**  **·**Potent natural antioxidant | Parkinson’s disease | Rotenone-treated PC12 and SH-SY5Y cells  **·**2 µg/mL rotenone and 2 µg/mL CPNP for 48 h | | **Improved:**  **·**Bcl-2/BAX ratio | **Reduced**:  **·**Cleaved caspase-3 and cleaved PARP expression in rotenone exposed cells | (Kundu et al., 2016) |
| 4 | SLNs | **Hydrodynamic diameter (nm)**  **·**172.10 ± 7.41 | **Thymoquinone (TQ):**  **·**Anti-inflammatory  **·**Neuroprotector  **·**Suppressor of oxidative stress neuropathy | Huntington’s disease | 3-NP-treated male albino rats  **·**10, 30, and 80 mg/kg b.w orally 1 h prior to 10 mg/kg b.w i.p. 3-NP for 14 days | | **Improved:**  **·**SDH enzyme activity | **Reduced:**  **·**Oxidative stress markers (LPO, protein carbonyls and NO) | (Ramachandran & Thangarajan, 2016) |
| 5 | Albumin-based NPs (R-FP-NP) | **Hydrodynamic diameter (nm)**  **·**284.4 ± 14.9 | **R-Flurbiprofen:**  **·**Depolarizes mitochondria  **·**Inhibitor of Ca^2+^ uptake even at low mM | Alzheimer’s disease | *In vivo:*  Adult male C57BL/6 mice  **·**2 mg/kg b.w i.n for 0.5, 1, 1.5, 2, and 4 h | *In vitro:*  CHO cells stably transfected with mouse Ab precursor protein 695 (CHO-APP695)  **·**25, 50, 100 and 200 µM for 48 h  **·**1 and 10 µM for 24 h (mitochondrial functioning studies) | **Improved:**  **·**Basal respiration  **·**Basal and ATP-linked OCR  **·**Proton leak  **·**Coupling efficiency values  **·**Reserve respiratory capacity  **·**Maximal mitochondrial respiration upon FCCP stimulation | **Reduced:**  **·**Non-mitochondrial respiration following rotenone + antimycin A addition | (Wong & Ho, 2017) |
| 6 | AuNPs | **Hydrodynamic diameter (nm)**  **·**20  **ZP (mV)**  **·**-30 | Not stated | Alzheimer’s disease | Intracerebroventricular-STZ injected Wistar male rats  **·**2.5 mg/kg b.w i.p 48 h following STZ injection | | **Induced:**  **·**Mitochondrial functionality  **·**Proton gradient by succinate | **Reduced:**  **·**Superoxide and NO_2_^-^ levels, and H_2_O_2_ levels indicating ROS reduction | (Muller et al., 2017) |
| 7 | SLNs | **Hydrodynamic diameter (nm)**  **·**286 ± 1.47  **ZP (mV)**  **·**-17.5±0.23 | **Resveratrol:**  **·**Antioxidant  **·**Anti-inflammatory  **·**Antiaging  **·**Neuroprotector | Vascular dementia (VaD) | Hypoxia-induced oxidative stress BCCAO male Sprague-Dawley (SD) rats  **·**10 mg RSV/kg b.w orally for 90 min | | **Improved:**  **·**Levels of GSH  **·**Redox ratio  **·**Nrf2 mRNA expression and protein levels (nuclear to cytoplasmic ratio)  **Rescued:**  **·**Mn-SOD activity | **Reduced:**  **·**Production of ROS and lipid peroxidation  **·**Mitochondrial GSSG and protein carbonyl levels | (Yadav et al., 2018) |
| 8 | PEG-PLGA NPs | **Hydrodynamic diameter (nm)**  **·**133 ± 17  **ZP (mV)**  **·**+55 ± 6.23 | **Curcumin:**  **·**Potent natural antioxidant | Cerebral ischemia-reperfusion (CIR) | CIR-induced female SD rats  **·**5 mg/kg b.w orally containing 200 µg Cur 24 h prior to CIR induction | | **Improved:**  **·**Mitochondrial membrane microviscosity  **·**Cytosolic Bax pool values  **·**Cyt *c* levels for the mitochondrial fractions  **Rescued:**  **·**Bcl-2 expression to normal cellular level  **·**iNOS and COX-2 expression levels | **Reduced:**  **·**ROS levels  **·**NADH oxidase activity  **·**Cyt *c* in cell cytosol  **·**Level of the apoptotic factor in CIR-induced rats  **Prevented:**  **·**CIR induced lipid peroxidation  **·**Mitochondrial Bax accumulation  **·**Activation of caspase-3 and -9 | (Mukherjee et al., 2019) |
| 9 | Fe_3_O_4_ magnetic NPs | **Hydrodynamic diameter (nm)**  **·**185  **ZP (mV)**  **·**-37.5 | **Curcumin:**  **·**Potent natural antioxidant | Schizophrenia (SCZ) | Cerebellum cells of ketamine-treated Wistar rats  **·**30 mg/kg b.w by lateral tail vein injection for 5 days after ketamine treatment | | **Improved:**  **·**Complex II activity (measured by SDH activity)  **·**MMP  **·**ATP levels | **Reduced:**  **·**ROS production in brain mitochondria  **·**Cyt *c* release | (Naserzadeh et al., 2018) |
| 10 | NPs synthesised using biocompatible polymers as the antisolvent (NRSV) | **Hydrodynamic diameter (nm)**  **·**50-200 | **Resveratrol:**  ·Antioxidant  ·Anti-inflammatory  ·Antiaging  ·Neuroprotector | Parkinson’s disease | Rotenone-treated male albino Wistar rats  **·**40 mg/kg b.w., p.o. for 35 days following 2 mg/kg b.w., s.c. rotenone | | **Rescued:**  **·**Complex I activity  **·**SOD, citrate synthase, and aconitase activities | **Reduced:**  **·**Rotenone-induced lipid peroxidation | (Palle & Neerati, 2018) |
| 11 | Cu_x_O NP clusters (NCs) | **Hydrodynamic diameter (nm)**  **·**65 ± 7 | Not stated | Parkinson’s disease | **·**MPP^+^-treated SHSY-5Y cells  **·**UVA-treated NIH-3T3 cells  **·**OxLDL-treated cultured PIE cells  **·**5 and 10 µg/mL | | **Improved:**  **·**Cell viability in all cellular models | **Reduced:**  **·**Caspase-3 protein expression in all cellular models | (Hao et al., 2019) |
| 12 | AuNPs associated with NAC | **Hydrodynamic diameter (nm)**  **·**20 | Not stated | Sepsis-induced brain injury | CLP-induced adult male Wister rats  **·**50 mg/kg b.w. s.c., immediately and 12 h after surgery | | **Improved:**  **·**Complex I activity | **Reduced:**  **·**Lipid peroxidation in the hippocampus and prefrontal cortex  **·**Protein carbonyls in the hippocampus | (Petronilho et al., 2020) |
| 13 | Palladium hydride (PdH) NPs | **Hydrodynamic diameter (nm)**  **·**30 | Not stated | Alzheimer’s disease | *In vivo:*  3×Tg-AD mice carrying human gene mutants APPswe, PS1M146V and tauP301L  **·**0.5, 1 and 2 mg/mL bilateral intracerebral injection | *In vitro:*  Over expressed human Swedish mutant APP695 cell lines (N2a-SW)  **·**0.5, 1 and 2 mg/mL for 24 h | *In vivo:*  **Reduced:**  **·**Aβ oligomers levels  **Rescued:**  **·**Drp1 and Mfn2 expression levels  **Improved:**  **·**COX IV expression | *In vitro:*  **Improved:**  **·**Basal respiration, ATP production, H^+^ proton leak and maximum respiration  **·**COX IV expression  **Rescued:**  **·**Drp1 and Mfn2 expression levels  **·**MMP  **Reduced:**  **·**Ca^2+^ level | (Zhang et al., 2019) |
| 14 | Cerium oxide NPs (CeONPs) | **Hydrodynamic diameter (nm)**  **·**10 | Not stated | Mild traumatic brain injury (mTBI) | *In vitro*:  Mild lateral fluid percussion brain injury induced adult male SD rats  **·**0.15, 0.25, 1.5 and 2.5 µg/g i.v post injury | *In vivo*:  Mixed organotypic neuronal cultures from 94A Cell Injury Controller neonatal rats  **·**CeONPs (1 nM–1 μM) 1 h post injury | *In vivo*:  **Improved:**  **·**SOD and catalase activity  **·**GSH/GSSG ratio  **·**NT levels to normal (product of free radical damage)  **Reduced:**  **·**LOOH levels (byproduct of lipid peroxidation) | *In vitro:*  **Restored:**  **·**Response to injury-induced glutamate signalling | (Bailey et al., 2020) |
| 15 | AuNPs | **Hydrodynamic diameter (nm)**  **·**3-5 | Not stated | Alzheimer’s disease | Aβ_1-42_-treated hNSCs  **·**10 ppm for 48 h | | **Improved:**  **·**PGC1α, NRF-1, and Tfam mRNA levels  **·**ATP levels and mitochondrial mass | **Normalized:**  **·**Max mitochondrial respiratory function  **·**Cyt *c* oxidase activity  **·**MMP and mitochondrial morphology | (Chiang et al., 2020) |
| 16 | AuNPs | **Hydrodynamic diameter (nm)**  **·**Not reported | Not stated | Alzheimer’s disease | OA-treated Wistar male rats  **·**2.5 mg/kg b.w. i.p every 48 h for 21 days | | **Rescued:**  **·**Mitochondrial activity (especially complex V) | **Reduced:**  **·**Succinate-induced H_2_O_2_ production | (dos Santos Tramontin et al., 2020) |
| 17 | Zinc Oxide (ZnO) NPs | **Hydrodynamic diameter (nm)**  **·**242.7 ± 3.897  **ZP (mV)**  **·**15.5 ± 0.6 | Not stated | Neuronal differentiation | NGF differentiated PC12 cells  **·**2-12 µg/mL for 24, 48, and 72 h | | **Improved:**  **·**Basal respiration | **Reduced:**  **·**Maximal respiration  **·**ATP production and spare respiratory capacity  **·**Proton leak | (Srivastava et al., 2020) |
| 18 | Nanocrystals (HstN) | **Hydrodynamic diameter (nm)**  **·**Not reported  **ZP (mV)**  **·**Not reported | **Hesperetin:**  **·**High antioxidant  **·**Radical scavenger | Alzheimer’s disease | SH-SY5Y cell line harbouring neuronal amyloid precursor protein (APP695)  **·**0.01-10 µM for 24 h | | **Improved:**  **·**Complex I & IV activity, OXPHOS, ETC, and leak respiration  **·**ATP levels  **·***CS*, *COX5A*, *NDUFV1*, and *ATP5F1D* mRNA levels  **·**Aβ_1-40_ expression | **Reduced:**  **·**Peroxidase and cyt *c* activity  **·**Small lowering effect on ROS | (Lukas Babylon et al., 2021) |
| 19 | Borneol modified octahedral palladium nanocomposites (Pd@PEG@Bor) | **Hydrodynamic diameter (nm)**  **·**Pd@PEG-COOH = 10.5  **·**Pd@PEG@Bor = 15.6  **ZP (mV)**  **·**Pd@PEG-COOH = -32.8  **·**Pd@PEG@Bor = -13.5 | Not stated | Alzheimer’s disease | *In vitro:*  **·**H_2_O_2_/Aβ -treated SH-SY5Y cells for ROS and MMP detection, respectively  **·**20 μg/mL for 24 h | *In vivo:*  **·**C57BL/6 and 3XTg-AD mice  **·**1 mg/kg b.w i.v for 4 h | **Recovered:**  **·**Low MMP in Aβ-treated SH-SY5Y cells | **Reduced:**  **·**OH^-^ levels at higher concentrations of Pd@PEG@Bor  **·**Aβ-induced cell apoptosis | (Jia et al., 2021) |
| 20 | Nanoemulsions | **Hydrodynamic diameter (nm)**  **·**20  **ZP (mV)**  **·** -14.8 | **Curcumin:**  **·**Potent natural antioxidant | Parkinson’s disease | Rotenone-treated Male Swiss Albino mice  **·**25 and 50 mg/kg b.w orally for 30 days with 1 mg/kg rotenone administered i.p on day 8 | | **Prevented:**  **·**Rotenone-induced inhibition of mitochondrial complex I activity | | (Ramires Júnior et al., 2021) |
| 21 | AuNPs | **Hydrodynamic diameter (nm)**  **·**20 | Not stated | Hypercholesterolemia | Adult Swiss mice fed a high cholesterol diet  **·**2.5 µg/kg by oral gavage every 2 days | | **Improved:**  **·**Prefrontal cortex complex I activity in both normal and high cholesterol diet | | (Rodrigues et al., 2021) |
| 22 | SLNs (GSE/DA-SLN) | **Hydrodynamic diameter (nm)**  **·**Blank SLN = 141±11  **·**GSE/DA-SLN= 184±34  **ZP (mV)**  **·**Blank SLN = -9.7±0.8  **·**GSE/DA-SLN = -2.7±0.2 | **Grape seed extract and dopamine:**  Antioxidants | Parkinson’s disease | OECs and 6-OHDA-induced SH-SY5Y cells  **·**200 µL of 75 µM DA for 24 h | | **Improved:**  **·**Antioxidant activity | | (Adriana Trapani et al., 2021) |
| 23 | Polydopamine NPs (PDNPs) | **Hydrodynamic diameter (nm)**  **·**296.8 ± 2.7  **ZP (mV)**  **·**−46.7 ± 0.3 | Not stated | Autosomal recessive spastic ataxia of Charlevoix-Saguenay (ARSACS) | TBH-treated ARSACS patient-derived fibroblasts & healthy fibroblasts  **·**0, 12.5, 25, 50, 100 and 250 μg/mL for 72 h | | **Recovered:**  **·**TBH-induced elongation loss of the mitochondria **Prevented:**  **·**Increment in apoptotic and necrotic cells  **·**TBH-induced fragmentation of the mitochondria  **·**TBH-induced mitochondrial damage through MMP stability | **Reduced:**  **·**Basal ROS | (Battaglini et al., 2022) |
| 24 | AuNPs | **Hydrodynamic diameter (nm)**  **·**5.13 ± 1.10  **ZP (mV)**  **·**−32 | **GSH:**  **·**Potent antioxidant  **·**Free radical scavenger and ROS balancer in the brain | Alzheimer’s disease | Aβ_1-42_-treated hNSCs  **·**0.25 mM in 1 mM GSH | | **Improved:**  **·**SOD, catalase and HO^-1^ activity  **·**PGC1α, NRF-1 and Tfam protein levels  **·**MMP  **Normalized:**  **·**Oxidative stress in Aβ-induced cells  **·**Release of cyt c from the mitochondria into the cytosol | **Reduced:**  **·**Superoxide production  **·**Cyt *c* oxidase activity **Rescued:**  **·**Aβ-induced ATP loss, D-loop and mitochondrial mass  **·**Basal respiration, ATP-linked respiration, and maximal respiration capacity  **·**PGC1α, NRF-1 and Tfam gene expression | (Chiang & Nicol, 2022) |
| 25 | PLGA-based NPs | **Hydrodynamic diameter (nm)**  **·**211.1  **ZP (mV)**  **·**−34.4 | **PINK1 siRNA:**  **·**Reduces mitophagy through reduction of *PINK1* mRNA levels | Photothrombosis-induced ischemic stroke (PTS) | RB-induced PTS murine model  **·**0–500 μg/ml injected intrathecally 2 days prior to RB-induction | | **Reduced:**  **·**Mitophagy and autophagy prior to PTS induction via the reduction of PINK1, LC3B, and lamp1 protein expression  **·**MMP depolarization | | (Choi et al., 2023) |
| 26 | β-cyclodextrin NPs (NSβ-CD) | Not stated | Not stated | Niemann-Pick type C1 (NPC) | Blood and urine samples from patients with NPC and fibroblasts derived from skin biopsies taken from patients with NPC  **·**45 μM of β-CD for 48 h | | **Reduced:**  **·**Mitochondrial superoxide production | | (Hammerschmidt et al., 2023) |
| 27 | Tween 80 coated PLGA NPs | **Hydrodynamic diameter (nm)**  **·**92 ± 7  **ZP (mV)**  **·**−14 ± 1 | **NONOates:**  **·**Mitochondrial bioenergetics initiator | Alzheimer’s disease | LPS-induced adult Swiss Abino mice  **·**2 mg/kg/dose i.v | | **Improved:**  **·**Cyt *c* oxidase enzyme activity  **·**ATP levels | | (Samir et al., 2023) |

3-NP: 3-Nitropropionic Acid; 6-OHDA: 6-hydroxydopamine; ATP: Adenine triphosphate; ATP5F1D: ATP synthase F1 subunit delta; BAX: Bcl-2-associated X-protein; Bcl-2: B-cell leukemia/lymphoma 2 protein; CLP: Cecal ligation and puncture; COX IV: Cytochrome c oxidase subunit IV; COX-2: Cyclooxygenase-2; COX5A: Cytochrome c oxidase subunit 5A; CS: Citrate synthase; Cyt a, b, c: Cytochrome a, b, c; Drp1: Dynamin related protein 1; ETC: Electron transfer chain; FCCP: Carbonylcyanide-p-trifluoromethoxyphenylhydrazone; GSH: Glutathione; GSSG: Oxidised glutathione; H_2_O_2_: Hydrogen Peroxide; iNOS: Nitric oxide synthase; lamp1: Lysosomal associated membrane protein 1; LC3B: Light chain 3 beta; LOOH: Lipid hydroperoxide; LPO: Lipid peroxidation; LPS: Lipopolysaccharide; MDA: Malondialdehyde; Mfn2: Mitofusin-2 protein; MMP: Mitochondrial membrane potential; Mn-SOD: Manganese superoxide dismutase; MPP^+^: 1-methyl-4-phenylpyridinium; mRNA: Messenger ribonucleic acid; Mt: Mitochondria; NADH: Nicotinamide adenine dinucleotide; NDUFV1: NADH:ubiquinone oxidoreductase core subunit V1; NGF: Nerve growth factor; NO: Nitric oxide; NO_2_^-^: Nitrite; NP(s): Nanoparticle(s); NRF1/2: Nuclear factor erythroid 2-related factor 1/2; NT: Nitrotyrosine; OCR: Oxygen consumption rate; OxLDL: Oxidised low-density lipoprotein; OXPHOS: Oxidative phosphorylation; PARP: Poly (ADP-ribose) polymerase; PF-68: Pluronic F-68; PGC1α: Peroxisome proliferator-activated receptor gamma coactivator 1-alpha; PINK1: PTEN induced kinase 1; RB: Retinoblastoma; R-FP: R-Flurbiprofen; ROS: Reactive oxygen species; SDH: Succinate dehydrogenase; SOD: Superoxide dismutase; STZ: Streptozotocin; TBH: Tert-butylhydroperoxide; Tfam: Mitochondrial transcription factor A; TPGS: Tocophersolan; UVA: Ultra-violet A; VIP: Vascular intestinal peptide

Supplementary Table S2**.** *In vitro* and *in vivo* studies of drug loaded mitochondria (mt)-targeted nanoparticles (NPs) and the effect on mt functioning in the literature

|  | Type of NP | Characteristics of NP | Drug loaded^[[3]](#footnote-3)^ | Method of mt targeting | Mt targeting identification | Brain disorder | Experimental model and treatment conditions^[[4]](#footnote-4)^ | | Effect of the NP treatment on Mt functioning | | Reference^[[5]](#footnote-5)^ | | |
| --- | --- | --- | --- | --- | --- | --- | --- | --- | --- | --- | --- | --- | --- |
| 1 | Ceria NPs | **Hydrodynamic diameter (nm)**  **·**Ceria NP: 3.0 ± 0.8  **·**TPP-Ceria-NP: 24 ± 2  **ζ-potential (ZP) (mV)**  **·**Ceria NP: -23  **·**TPP-Ceria-NP: +45 | **Ceria:**  Scavenger for ROS by shuttling between Ce^3+^ and Ce^4+^ oxidative states | **·**TPP^+^  **·**EDC coupling | Nuclear magnetic resonance (H^1^ NMR) | Alzheimer’s disease | *In vivo:*  5XFAD transgenic AD mice  **·**0.125, 0.25, 0.5, and 1 mM for 24 h | *In vitro:*  Aβ-induced SH-SY5Y cells  **·**0.125-1 mM for 24 h | *In vivo:*  **Recovered:**  Severe cristae disruptions in 5XFAD mice  **Reduced:**  4-HNE in 5XFAD mice | *In vitro:*  **Prevented:**  Aβ-induced mitochondrial ROS | | (Kwon et al., 2016) | |
| 2 | 4 hydroxyl-terminated PAMAM dendrimers | **Hydrodynamic diameter (nm)**  **·**D-OH: 4.4±0.2  **·**TPP-D-NAC: 7.5±0.2  **ZP (mV)**  **·**D-OH: -4.5±0.6  **·**TPP-D-NAC: +16.9±0.4 | **D-NAC:**  **·**Antioxidant  **·**Anti-inflammatory | **·**TPP^+^  **·**Amide linkages | H^1^ NMR | Traumatic brain injury  (TBI) | Human macrophages, murine microglia and paediatric TBI model  **·**50 µg/ml for 24 h | | **Rescued:**  **·**Mitochondrial O_2_^-^levels back to within 10% of healthy control cells  **·**MMP to approximately 90% of healthy cell levels | **Reduced:**  O_2_^-^levels compared to H_2_O_2_ stimulated cells  **Preserved:**  Metabolic activity of H_2_O_2_ stimulated cells | | (Sharma et al., 2018) | |
| 3 | Red blood cell (RBC) membrane-camouflaged human serum albumin NPs  (T807/TPP-RBC) | **Hydrodynamic diameter (nm)**  **·**RBC-NP: 102.62±0.59  **·**T807/TPP-RBC-NP: 112.13±0.74  **ZP (mV)**  **·**RBC-NP: -39.3±0.9  **·**T807/TPP-RBC-NP: +10.9±0.5 | **Curcumin:**  **·**Potent natural antioxidant  **GSH:**  **·**Potent antioxidant  **·**Free radical scavenger and ROS balancer in the brain | **·**TPP^+^  **·**NH_2_-carboxy coupling reaction | Molecular shifts in MALDI-TOF MS | Alzheimer’s disease | *In vivo*:  Male ICR mice and Sprague-Dawley (SD) rats treated with OA  **·**2 mg/kg b.w T807/TPP-RBC-NPs i.v.  daily for 10 days | *In vitro*:  RAW264.7, BMECs and HT22 cells  **·**5 µM T807/TPP-RBC-NPs for 12 h | **Reduced:**  **·**Mitochondrial ROS levels *in vivo* and *in vitro*  **·**SOD, ϒ -GT, MDA and H_2_O_2_ levels *in vitro*  **·**Cell apoptosis rate *in vitro* | | | (Gao et al., 2020) | |
| 4 | RBC membrane-coated nanostructured lipid particles  (TPP-RSV-RBC) | **Hydrodynamic diameter (nm)**  **·**RSV-RBC:  148.51±0.94  **·**TPP-RSV-RBC: 150.71±0.31  **ZP (mV)**  **·**RSV-RBC:  -23.6±0.4  **·**TPP-RSV-RBC: +17.3±0.1 | **Resveratrol (RSV):**  **·**Antioxidant | **·**TPP^+^  **·**NH_2_-carboxy coupling reaction | MALDI-TOF MS and H^1^ NMR | Alzheimer’s disease | *In vivo*:  **·**Male ICR mice, SD rats, and APP/PS1 mice  **·**2 mg/kg b.w NPs i.v. every 2 days for 30 days | *In vitro:*  **·**Peritoneal macrophages and primary astrocytes isolated from C57BL/6J mice, Aβ-treated bEnd.3 and HTT2 cells  **·**5 µg/mL for 4 h | *In vivo:*  **Reduced:**  Mitochondrial ROS in APP/PS1 mice | *In vitro:*  **Reduced:**  Mitochondrial ROS, MnSOD, MDA and cell apoptosis in Aβ-treated HTT2 cells | | | (Han et al., 2020) |
| 5 | PLGA-b-PEG-TPP | **Hydrodynamic diameter (nm)**  **·** 50-70  **ZP (mV)**  **·**+20-29 | ARVs/cART | **·**TPP^+^  **·**NHS-amino coupling reaction | Not stated | HIV-associated neurocognitive disorders | *In vivo:*  EcoHIV and Meth infected Balb/c albino female mice and C57BL/6 male mice  ·5 mg/kg i.v. twice weekly for 2 weeks | *In vitro:*  **·**HMC3, CRL-3304, and nHA cells and isolated astrocytes and neuronal cells  **·**1 µM for 24 h | **Rescued:**  Mitochondrial ROS in HIV+Meth induced microglia cells  **Improved:**  ATP production | **Reduced:**  **·**O_2_^-^ levels  **·**Oxidative stress (measured by the mRNA levels of the ROS markers GCLC, GCLM, and GPX7) in HIV+Meth induced cells  **·**ROS levels in the mice | | | (Surnar et al., 2021) |
| 6 | AuNPs (PEG-AuNPs@Hyp) | **Hydrodynamic diameter (nm)**  **·**AuNPs: ~30±0.2  **·**PEG-AuNPs@Hyp: ~50±1.2  **ZP (mV)**  **·**AuNPs: -23.8±2  **·**PEG-AuNPs@Hyp:  -20.8±2 | **Au:**  **·**Anti-inflammatory  **·**Antioxidant | Hypericin | Crystallographic planes identified with high resolution TEM | Glioblastoma | HEK293T, LN18 and C6 cell lines  **·**0.25–5 μM for 48 h | | **Induced:**  **·**MMP depolarisation and loss  **·**Mitochondria intracellular ROS apoptosis  **·**ROS-induced glioma cell death and overexpression of caspase-3, therefore, abrogating GBM proliferation | | | (Kaundal et al., 2022) | |
| 7 | Citraconylation modified PEG- (FGL-NP(Cit)/HNSS) | **Hydrodynamic diameter (nm)**  **·**NP(Cit)/HNSS:  115.6±0.5  **·**FGL-NP(Cit)/HNSS: 127.7±0.9  **ZP (mV)**  **·**NP(Cit)/HNSS:  -31.6±1.3  **·**FGL-NP(Cit)/HNSS:  -26.4±2.7 | Hybrid peptide HNSS | SS31 targeting moiety and humanin | Confocal microscopy | Alzheimer’s disease | 3xTg-AD transgenic mice with mutated human APP(Swe), tau(P301L), and PS1(M146 V) genes  **·**0.3 mg/kg/d i.v. for 28 days | | **Prevented:**  Intracellular and mitochondrial ROS production  **Rescued:**  **·**GSH and SOD activity  **·**Aβ_25−35_-induced impaired mitochondrial function  **·**Morphology of mitochondria  **·**Dynamic imbalance of MFN1 and DRP1 proteins | **Improved:**  **·**p-STAT3, cyt c MTCO1 (subunit of complex IV protein) and MnSOD expression levels  **·**p-STAT3/STAT3 ratio  **·**TAOC and SOD activity  **·**PGC-1α mRNA level  **Reduced:**  MDA levels | | (Qian et al., 2022) | |
| 8 | Tetrahedral DNA framework-based NPs (TDFNs) | **Hydrodynamic diameter (nm)**  **·**TDFNs: 7.58  **ZP (mV)**  **·**TDFNs: -10.94 | Functional antisense oligonucleotide (ASO) for both AD diagnosis and gene silencing | **·**TPP^+^  **·**TPP-COOH addition | Cell imaging | Alzheimer’s disease | *In vivo:*  **·**3 × Tg-AD model mice  **·**1 µM i.v. every 2 days | *In vitro:*  **·**Aβ-treated SH-SY5Y cells  **·**HEK293/TAU cells  **·**Gradient concentration for 24 h | *In vivo:*  **Rescued:**  Neuronal mitochondria by ASO-induced miRNA-24a knock down | *In vitro:*  **Rescued:**  Apoptosis of Aβ-treated SH-SY5Y cells  **Improved:**  MMP in Aβ-treated SH-SY5Y cells | | (Li et al., 2023) | |
| 9 | Cu_2_-xSe-based (CSP) NPs (CSCCT NPs) | **Hydrodynamic diameter (nm)**  **·**CSP NPs: 2.4±0.4  **·**CSCCT NPs: 47.6±7.5  **ZP (mV)**  **·**CSP NPs: -23  **·**CSCCT NPs: -33.8 | **Curcumin:**  **·**Potent natural antioxidant | **·**TPP^+^  **·**DSPE-PEG_2000_-TPP addition | Colocalization studies | Parkinson’s disease | *In vivo:*  **·**MPTP and US-treated C57BL/6 mice  **·**5 mg/kg i.v. | *In vitro:*  **·**RAW 264.7 and MPTP induced SH-SY5Y cell lines  **·**25 µM for 2 h | *In vivo:*  **Improved:**  PGC-1𝛼, SIRT1,  TFAM, and Gpx4 expression  **Rescued:**  Ndufs1 expression and Gpx4 enzyme activity in mitochondria | *In vitro:*  **Improved**:  **·**PGC-1𝛼 deacetylation and PGC-1𝛼-induced PPAR𝛾, NRF1, and TFAM mRNA levels  **·**NAD^+^/NADH ratio, SIRT1 activation and mtDNA copy number  **Rescued:**  Gpx4, CAT, and SOD2 levels, GSH/GSSG ratio and Mitochondrial cristae  **Prevented:**  MPP^+^-induced mitochondrial ROS production and reduction of MMP  **Reduced:**  Cyt *c* release | | (Zheng et al., 2023) | |

4-HNE: 4-Hydroxynonenal; 5XFAD: Mice that bear five Familial Alzheimer’s Disease-linked mutated genes; ARVs/cART: Antiretrovials/combined antiretrovirals; ASO: Antisense oligonucleotide; ATP: Adenosine triphosphate; Au: Gold; Aβ: Amyloid beta peptide; CAT: Catalase; Cu_2_-xSe: Copper chalcogenide; cyt: Cytochrome; DNA: Deoxyribonucleic acid; D-NAC: Dendrimer conjugated N-acetyl cysteine; DRP1: Dynamin related protein 1;GCLC: Glutamate-cysteine ligase catalytic subunit; GCMC: Glutamate-cysteine ligase modifier subunit; Gpx4: Glutathione peroxidase 4; GPX7: Glutathione peroxidase 7; GSH; Glutathione; GSSG: Oxidised glutathione; H_2_O_2_: Hydrogen peroxide; HIV: Human immunodeficiency virus; MDA: Malonaldehyde; Meth: Methamphetamine; MFN1; Mitofusin 1; MMP: Mitochondrial membrane potential; MnSOD: Manganese superoxide dismutase; MPTP: 1-methyl-4-phenyl-1,2,3,6-tetrahydropyridine; mRNA: Messenger ribonucleic acid; MTCO1: Mitochondrially Encoded Cytochrome C Oxidase I; mtDNA: Mitochondrial DNA; NAD^+^: Nicotinamide adenine dinucleotide; NADH: Nicotinamide adenine dinucleotide + hydrogen; Ndufs1: NADH:ubiquinone oxidoreductase core subunit S1; NP(s): Nanoparticle(s); NRF1: Nuclear respiratory factor 1; O_2_^-^: Superoxide anion; OA: Okadaic acid; PAMAM: Polyamidoamine; PGC1α: Peroxisome proliferator-activated receptor gamma coactivator 1-alpha; PLGA-b-PEG: Poly(D,L-lactic-co-glycolic acid)-block-polyethylene glycol); PPAR𝛾: Peroxisome proliferator-activated receptor gamma; (p)STAT3: (Phosphorylated) transducer and activator of transcription-3; ROS: Reactive oxygen species; SIRT1: Sirtuin 1; SOD: Superoxide dismutase; SOD2: Superoxide dismutase 2; SS31: Szeto-Schiller peptide 31; TAOC: Total antioxidant capacity; Tfam: Mitochondrial transcription factor A; TPP^+^: Triphenylphosphonium cation; US: Ultrasonic; ϒ-GT: Gamma-glutamyl transpeptidase

# References

Abdel-Mageed, H. M., AbuelEzz, N. Z., Radwan, R. A., & Mohamed, S. A. (2021). Nanoparticles in nanomedicine: a comprehensive updated review on current status, challenges and emerging opportunities [Article]. *Journal of Microencapsulation*, *38*(6), 414–436. https://doi.org/10.1080/02652048.2021.1942275

Adriana Trapani, Lorenzo Guerra, Filomena Corbo, Stefano Castellani, Enrico Sanna, Loredana Capobianco, Anna Grazia Monteduro, Daniela Erminia Manno, Delia Mandracchia, Sante Di Gioia, & Massimo Conese. (2021). Cyto/Biocompatibility of Dopamine Combined with the Antioxidant Grape Seed-Derived Polyphenol Compounds in Solid Lipid Nanoparticles [Article]. *Molecules (Basel, Switzerland)*, *26*(4), 916. https://doi.org/10.3390/molecules26040916

Aryal, S., Park, H., Leary, J. F., & Key, J. (2019). Top-down fabrication-based nano/microparticles for molecular imaging and drug delivery [Article]. *International Journal of Nanomedicine*, *14*, 6631–6644. https://doi.org/10.2147/ijn.s212037

Bailey, Z. S., Nilson, E., Bates, J. A., Oyalowo, A., Hockey, K. S., Sajja, V. S. S. S., Thorpe, C., Rogers, H., Dunn, B., Frey, A. S., Billings, M. J., Sholar, C. A., Hermundstad, A., Kumar, C., Vandevord, P. J., & Rzigalinski, B. A. (2020). Cerium Oxide Nanoparticles Improve Outcome after In Vitro and In Vivo Mild Traumatic Brain Injury. *Journal of Neurotrauma*, *37*(12), 1452–1462. https://doi.org/10.1089/neu.2016.4644

Battaglini, M., Carmignani, A., Martinelli, C., Colica, J., Marino, A., Doccini, S., Mollo, V., Santoro, F., Bartolucci, M., Petretto, A., Santorelli, F. M., & Ciofani, G. (2022). In vitro study of polydopamine nanoparticles as protective antioxidant agents in fibroblasts derived from ARSACS patients [Article]. *Biomaterials Science*, *10*(14), 3770–3792. https://doi.org/10.1039/d2bm00729k

Brentnall, M., Rodriguez-Menocal, L., De Guevara, R. L., Cepero, E., & Boise, L. H. (2013). Caspase-9, caspase-3 and caspase-7 have distinct roles during intrinsic apoptosis. *BMC Cell Biology*, *14*(1), 1–9. https://doi.org/10.1186/1471-2121-14-32

Chiang, M.-C., & Nicol, C. J. B. (2022). GSH-AuNP anti-oxidative stress, ER stress and mitochondrial dysfunction in amyloid-beta peptide-treated human neural stem cells [Article]. *Free Radical Biology & Medicine*, *187*, 185–201. https://doi.org/10.1016/j.freeradbiomed.2022.05.025

Chiang, M.-C., Nicol, C. J. B., Cheng, Y.-C., Yen, C., Lin, C.-H., Chen, S.-J., & Huang, R.-N. (2020). Nanogold Neuroprotection in Human Neural Stem Cells Against Amyloid-beta-induced Mitochondrial Dysfunction [Article]. *Neuroscience*, *435*, 44–57. https://doi.org/10.1016/j.neuroscience.2020.03.040

Choi, S. G., Shin, J., Lee, K. Y., Park, H., Kim, S. I., Yi, Y. Y., Kim, D. W., Song, H., & Shin, H. J. (2023). PINK1 siRNA‐loaded poly(lactic‐co‐glycolic acid) nanoparticles provide neuroprotection in a mouse model of photothrombosis‐induced ischemic stroke [Article]. *Glia*, *71*(5), 1294–1310. https://doi.org/10.1002/glia.24339

dos Santos Tramontin, N., da Silva, S., Arruda, R., Ugioni, K. S., Canteiro, P. B., de Bem Silveira, G., Mendes, C., Silveira, P. C. L., & Muller, A. P. (2020). Gold Nanoparticles Treatment Reverses Brain Damage in Alzheimer’s Disease Model [Article]. *Molecular Neurobiology*, *57*(2), 926–936. https://doi.org/10.1007/s12035-019-01780-w

Gao, C., Wang, Y., Sun, J., Han, Y., Gong, W., Li, Y., Feng, Y., Wang, H., Yang, M., Li, Z., Yang, Y., & Gao, C. (2020). Neuronal mitochondria-targeted delivery of curcumin by biomimetic engineered nanosystems in Alzheimer’s disease mice [Article]. *Acta Biomaterialia*, *108*, 285–299. https://doi.org/10.1016/j.actbio.2020.03.029

Hammerschmidt, T. G., Donida, B., Raabe, M., Faverzani, J. L., de Fátima Lopes, F., Machado, A. Z., Kessler, R. G., Reinhardt, L. S., Poletto, F., Moura, D. J., & Vargas, C. R. (2023). Evidence of redox imbalance and mitochondrial dysfunction in Niemann-Pick type C 1 patients: the in vitro effect of combined therapy with antioxidants and β‐cyclodextrin nanoparticles [Article]. *Metabolic Brain Disease*, *38*(2), 507–518. https://doi.org/10.1007/s11011-022-01128-9

Han, Y., Chu, X., Cui, L., Fu, S., Gao, C., Li, Y., & Sun, B. (2020). Neuronal mitochondria-targeted therapy for Alzheimer’s disease by systemic delivery of resveratrol using dual-modified novel biomimetic nanosystems [Article]. *Drug Delivery*, *27*(1), 502–518. https://doi.org/10.1080/10717544.2020.1745328

Hao, C., Qu, A., Xu, L., Sun, M., Zhang, H., Xu, C., & Kuang, H. (2019). Chiral Molecule-mediated Porous Cu x O Nanoparticle Clusters with Antioxidation Activity for Ameliorating Parkinson’s Disease [Article]. *Journal of the American Chemical Society*, *141*(2), 1091–1099. https://doi.org/10.1021/jacs.8b11856

Hillen, A. E. J., & Heine, V. M. (2020). Glutamate Carrier Involvement in Mitochondrial Dysfunctioning in the Brain White Matter. *Frontiers in Molecular Biosciences*, *7*, 551748. https://doi.org/10.3389/FMOLB.2020.00151

Hoshyar, N., Gray, S., Han, H., & Bao, G. (2016). The effect of nanoparticle size on in vivo pharmacokinetics and cellular interaction. *Nanomedicine*, *11*(6), 673. https://doi.org/10.2217/NNM.16.5

Jia, Z., Yuan, X., Wei, J., Guo, X., Gong, Y., Li, J., Zhou, H., Zhang, L., & Liu, J. (2021). A Functionalized Octahedral Palladium Nanozyme as a Radical Scavenger for Ameliorating Alzheimer’s Disease [Article]. *ACS Applied Materials & Interfaces*, *13*(42), 49602–49613. https://doi.org/10.1021/acsami.1c06687

Joudeh, N., & Linke, D. (2022). Nanoparticle classification, physicochemical properties, characterization, and applications: a comprehensive review for biologists [Article]. *Journal of Nanobiotechnology*, *20*(1), 262–262. https://doi.org/10.1186/s12951-022-01477-8

Kaundal, B., Karmakar, S., & Roy Choudhury, S. (2022). Mitochondria-targeting nano therapy altering IDH2-mediated EZH2/EZH1 interaction as precise epigenetic regulation in glioblastoma [Article]. *Biomaterials Science*, *1*(18), 531–5317. https://doi.org/10.1039/d1bm02006d

Kundu, P., Das, M., Tripathy, K., & Sahoo, S. K. (2016). Delivery of Dual Drug Loaded Lipid Based Nanoparticles across the Blood-Brain Barrier Impart Enhanced Neuroprotection in a Rotenone Induced Mouse Model of Parkinson’s Disease. *ACS Chemical Neuroscience*, *7*(12), 1658–1670. https://doi.org/10.1021/ACSCHEMNEURO.6B00207

Kwon, H. J., Cha, M.-Y., Kim, D., Kim, D. K., Soh, M., Shin, K., Hyeon, T., & Mook-Jung, I. (2016). Mitochondria-Targeting Ceria Nanoparticles as Antioxidants for Alzheimer’s Disease [Article]. *ACS Nano*, *10*(2), 2860–2870. https://doi.org/10.1021/acsnano.5b08045

Li, W., Peng, X., Mei, X., Dong, M., Li, Y., & Dong, H. (2023). Multifunctional DNA Tetrahedron for Alzheimer’s Disease Mitochondria-Targeted Therapy by MicroRNA Regulation [Article]. *ACS Applied Materials & Interfaces*, *15*(19), 22977–22984. https://doi.org/10.1021/acsami.3c03181

Lukas Babylon, Rekha Grewal, Pascal-L. Stahr, Ralph W. Eckert, Cornelia M. Keck, & Gunter P. Eckert. (2021). Hesperetin Nanocrystals Improve Mitochondrial Function in a Cell Model of Early Alzheimer Disease [Article]. *Antioxidants*, *10*(7), 1003. https://doi.org/10.3390/antiox10071003

Mukherjee, A., Sarkar, S., Jana, S., Swarnakar, S., & Das, N. (2019). Neuro-protective role of nanocapsulated curcumin against cerebral ischemia-reperfusion induced oxidative injury [Article]. *Brain Research*, *1704*, 164–173. https://doi.org/10.1016/j.brainres.2018.10.016

Muller, A. P., Ferreira, G. K., Pires, A. J., de Bem Silveira, G., de Souza, D. L., Brandolfi, J. de A., de Souza, C. T., Paula, M. M. S., & Silveira, P. C. L. (2017). Gold nanoparticles prevent cognitive deficits, oxidative stress and inflammation in a rat model of sporadic dementia of Alzheimer’s type [Article]. *Materials Science & Engineering C*, *77*, 476–483. https://doi.org/10.1016/j.msec.2017.03.283

Najahi-Missaoui, W., Arnold, R. D., & Cummings, B. S. (2020). Safe Nanoparticles: Are We There Yet? [Article]. *International Journal of Molecular Sciences*, *22*(1), 385. https://doi.org/10.3390/ijms22010385

Naserzadeh, P., Hafez, A. A., Abdorahim, M., Abdollahifar, M. A., Shabani, R., Peirovi, H., Simchi, A., & Ashtari, K. (2018). Curcumin loading potentiates the neuroprotective efficacy of Fe3O4 magnetic nanoparticles in cerebellum cells of schizophrenic rats [Article]. *Biomedicine & Pharmacotherapy*, *108*, 1244–1252. https://doi.org/10.1016/j.biopha.2018.09.106

Palle, S., & Neerati, P. (2018). Improved neuroprotective effect of resveratrol nanoparticles as evinced by abrogation of rotenone-induced behavioral deficits and oxidative and mitochondrial dysfunctions in rat model of Parkinson’s disease [Article]. *Naunyn-Schmiedeberg’s Archives of Pharmacology*, *391*(4), 445–453. https://doi.org/10.1007/s00210-018-1474-8

Petronilho, F., Tenfen, L., Della Giustina, A., Joaquim, L., Novochadlo, M., de Oliveira Junior, A. N., Bagio, E., Goldim, M. P. de S., de Carli, R. J., Bonfante, S. R. S. de A., Metzker, K. L. L., Muttini, S., dos Santos, T. M., de Oliveira, M. P., Engel, N. A., Rezin, G. T., Kanis, L. A., & Barichello, T. (2020). Gold nanoparticles potentiates N-acetylcysteine effects on neurochemicals alterations in rats after polymicrobial sepsis [Article]. *Journal of Drug Targeting*, *28*(4), 428–436. https://doi.org/10.1080/1061186X.2019.1678168

Qian, K., Bao, X., Li, Y., Wang, P., Guo, Q., Yang, P., Xu, S., Yu, F., Meng, R., Cheng, Y., Sheng, D., Cao, J., Xu, M., Wu, J., Wang, T., Wang, Y., Xie, Q., Lu, W., & Zhang, Q. (2022). Cholinergic Neuron Targeting Nanosystem Delivering Hybrid Peptide for Combinatorial Mitochondrial Therapy in Alzheimer’s Disease [Article]. *ACS Nano*, *16*(7), 11455–11472. https://doi.org/10.1021/acsnano.2c05795

Ramachandran, S., & Thangarajan, S. (2016). A novel therapeutic application of solid lipid nanoparticles encapsulated thymoquinone (TQ-SLNs) on 3-nitroproponic acid induced Huntington’s disease-like symptoms in wistar rats. *Chemico-Biological Interactions*, *256*, 25–36. https://doi.org/10.1016/J.CBI.2016.05.020

Ramires Júnior, O. V., Alves, B. da S., Barros, P. A. B., Rodrigues, J. L., Ferreira, S. P., Monteiro, L. K. S., Araújo, G. de M. S., Fernandes, S. S., Vaz, G. R., Dora, C. L., & Hort, M. A. (2021). Nanoemulsion Improves the Neuroprotective Effects of Curcumin in an Experimental Model of Parkinson’s Disease [Article]. *Neurotoxicity Research*, *39*(3), 787–799. https://doi.org/10.1007/s12640-021-00362-w

Rodrigues, M. S., de Paula, G. C., Duarte, M. B., de Rezende, V. L., Possato, J. C., Farias, H. R., Medeiros, E. B., Feuser, P. E., Streck, E. L., de Ávila, R. A. M., Bast, R. K. S. S., Budni, J., de Bem, A. F., Silveira, P. C. L., & de Oliveira, J. (2021). Nanotechnology as a therapeutic strategy to prevent neuropsychomotor alterations associated with hypercholesterolemia [Article]. *Colloids and Surfaces, B, Biointerfaces*, *201*, 111608. https://doi.org/10.1016/j.colsurfb.2021.111608

Samir, M., Abdelkader, R. M., Boushehri, M. S., Mansour, S., Lamprecht, A., & Tammam, S. N. (2023). Enhancement of mitochondrial function using NO releasing nanoparticles; a potential approach for therapy of Alzheimer’s disease [Article]. *European Journal of Pharmaceutics and Biopharmaceutics*, *184*, 16–24. https://doi.org/10.1016/j.ejpb.2023.01.006

Sandhir, R., Yadav, A., Mehrotra, A., Sunkaria, A., Singh, A., & Sharma, S. (2014). Curcumin nanoparticles attenuate neurochemical and neurobehavioral deficits in experimental model of Huntington’s disease. *NeuroMolecular Medicine*, *16*(1), 106–118. https://doi.org/10.1007/S12017-013-8261-Y

Sharma, A., Liaw, K., Sharma, R., Zhang, Z., Kannan, S., & Kannan, R. M. (2018). Targeting Mitochondrial Dysfunction and Oxidative Stress in Activated Microglia using Dendrimer-Based Therapeutics. *International Publisher Theranostics*, *8*(20), 20. https://doi.org/10.7150/thno.29039

Singh, R., & Lillard, J. W. (2009). Nanoparticle-based targeted drug delivery. *Experimental and Molecular Pathology*, *86*(3), 215. https://doi.org/10.1016/J.YEXMP.2008.12.004

Srivastava, A. K., Yadav, S. S., Mishra, S., Yadav, S. K., Parmar, D., & Yadav, S. (2020). A combined microRNA and proteome profiling to investigate the effect of ZnO nanoparticles on neuronal cells. *Nanotoxicology*, *14*(6), 757–773. https://doi.org/10.1080/17435390.2020.1759726

Surnar, B., Shah, A. S., Park, M., Kalathil, A. A., Kamran, M. Z., Ramirez Jaime, R., Toborek, M., Nair, M., Kolishetti, N., & Dhar, S. (2021). Brain-Accumulating Nanoparticles for Assisting Astrocytes to Reduce Human Immunodeficiency Virus and Drug Abuse-Induced Neuroinflammation and Oxidative Stress [Article]. *ACS Nano*, *15*(10), 15741–15753. https://doi.org/10.1021/acsnano.0c09553

Wong, L. R., & Ho, P. C. (2017). Role of serum albumin as a nanoparticulate carrier for nose-to-brain delivery of R-flurbiprofen: implications for the treatment of Alzheimer’s disease. *Journal of Pharmacy and Pharmacology*, *70*(1), 59–69. https://doi.org/10.1111/JPHP.12836

Xu, Z. R., Wang, W. F., Liang, X. F., Liu, Z. H., Liu, Y., Lin, L., & Zhu, X. (2015). Protective Effects of Poly (butyl) Cyanoacrylate Nanoparticles Containing Vasoactive Intestinal Peptide Against 6-Hydroxydopamine-Induced Neurotoxicity In Vitro. *Journal of Molecular Neuroscience*, *55*(4), 854–864. https://doi.org/10.1007/S12031-014-0438-9

Yadav, A., Sunkaria, A., Singhal, N., & Sandhir, R. (2018). Resveratrol loaded solid lipid nanoparticles attenuate mitochondrial oxidative stress in vascular dementia by activating Nrf2/HO-1 pathway [Article]. *Neurochemistry International*, *112*, 239–254. https://doi.org/10.1016/j.neuint.2017.08.001

Zashikhina, N., Gladnev, S., Sharoyko, V., Korzhikov-Vlakh, V., Korzhikova-Vlakh, E., & Tennikova, T. (2023). Synthesis and Characterization of Nanoparticle-Based Dexamethasone-Polypeptide Conjugates as Potential Intravitreal Delivery Systems [Article]. *International Journal of Molecular Sciences*, *24*(4), 3702. https://doi.org/10.3390/ijms24043702

Zhang, L., Zhao, P., Yue, C., Jin, Z., Liu, Q., Du, X., & He, Q. (2019). Sustained release of bioactive hydrogen by Pd hydride nanoparticles overcomes Alzheimer’s disease [Article]. *Biomaterials*, *197*, 393–404. https://doi.org/10.1016/j.biomaterials.2019.01.037

Zheng, Q., Liu, H., Zhang, H., Han, Y., Yuan, J., Wang, T., Gao, Y., & Li, Z. (2023). Ameliorating Mitochondrial Dysfunction of Neurons by Biomimetic Targeting Nanoparticles Mediated Mitochondrial Biogenesis to Boost the Therapy of Parkinson’s Disease [Article]. *Advanced Science*, *10*(22), e2300758-n/a. https://doi.org/10.1002/advs.202300758

Zielinska, A., Carreiró, F., Oliveira, A. M., Neves, A., Pires, B., Nagasamy Venkatesh, D., Durazzo, A., Lucarini, M., Eder, P., Silva, A. M., Santini, A., & Souto, E. B. (2020). Polymeric Nanoparticles: Production, Characterization, Toxicology and Ecotoxicology. *Molecules 2020, Vol. 25, Page 3731*, *25*(16), 3731. https://doi.org/10.3390/MOLECULES25163731

1. Properties mentioned relate only to mitochondrial functioning [↑](#footnote-ref-1)
2. Articles arranged in chronological order [↑](#footnote-ref-2)
3. Properties mentioned relate only to mitochondrial functioning benefit [↑](#footnote-ref-3)
4. Experimental models relating only to mitochondrial studies are mentioned [↑](#footnote-ref-4)
5. Articles arranged in chronological order [↑](#footnote-ref-5)
